# Complete Mitogenomes of *Polypedates* Tree Frogs Unveil Gene Rearrangement and Concerted Evolution within Rhacophoridae

**DOI:** 10.3390/ani12182449

**Published:** 2022-09-16

**Authors:** Lin Cui, An Huang, Zhi He, Lisha Ao, Fei Ge, Xiaolan Fan, Bo Zeng, Mingyao Yang, Deying Yang, Qingyong Ni, Yan Li, Yongfang Yao, Huailiang Xu, Jiandong Yang, Zhimin Wei, Tongqing Li, Taiming Yan, Mingwang Zhang

**Affiliations:** 1 Key Laboratory of Livestock and Poultry Multi-omics, Ministry of Agriculture and Rural Affairs, College of Animal Science and Technology, Sichuan Agricultural University, Chengdu 611130, China; 2 Farm Animal Genetic Resources Exploration and Innovation Key Laboratory of Sichuan Province, Sichuan Agricultural University, Chengdu 611130, China; 3 College of Life Science, Sichuan Agricultural University, Ya’an 625014, China; 4 Institute of Millet Crops, Hebei Academy of Agriculture and Forestry Sciences, Shijiazhuang 050051, China; 5 Hebei Fisheries Technology Extension Center, Shijiazhuang 050051, China

**Keywords:** mitogenome, gene rearrangement, phylogenetic analysis, Rhacophoridae, *Polypedates*

## Abstract

**Simple Summary:**

Duplicated control regions have been reported several times in the tree frog family Rhacophoridae, and previous studies have mostly relied on sequence analysis to reconstruct their evolution. This is the first study to employ a phylogenetic method to demonstrate the existence of concerted and parallel evolution succinctly and intuitively in the duplicated control regions of the family Rhacophoridae. Phylogenetic relationships were also used to illustrate the parallel evolution of *ATP8* loss of function in the genus *Polypedates*. In general, this study elucidated the evolutionary patterns and pathways of mitochondrial gene rearrangement of the family Rhacophoridae from a phylogenetic perspective, which aids in understanding the evolutionary history of this fascinating tree frog taxon from a molecular evolution standpoint.

**Abstract:**

New developments in sequencing technology and nucleotide analysis have allowed us to make great advances in reconstructing anuran phylogeny. As a clade of representative amphibians that have radiated from aquatic to arboreal habitats, our understanding of the systematic status and molecular biology of rhacophorid tree frogs is still limited. We determined two new mitogenomes for the genus *Polypedates* (Rhacophoridae): *P. impresus* and *P. mutus*. We conducted comparative and phylogenetic analyses using our data and seven other rhacophorid mitogenomes. The mitogenomes of the genera *Polypedates*, *Buergeria,* and *Zhangixalus* were almost identical, except that the *ATP8* gene in *Polypedates* had become a non-coding region; *Buergeria* maintained the legacy “LTPF” tRNA gene cluster compared to the novel “TLPF” order in the other two genera; and *B. buergeri* and *Z. dennysi* had no control region (CR) duplication. The resulting phylogenetic relationship supporting the above gene rearrangement pathway suggested parallel evolution of *ATP8* gene loss of function (LoF) in *Polypedates* and CR duplication with concerted evolution of paralogous CRs in rhacophorids. Finally, conflicting topologies in the phylograms of 185 species reflected the advantages of phylogenetic analyses using multiple loci.

## 1. Introduction

Vertebrate mitochondrial genomes (mitogenomes) are generally fixed with 37 genes: two ribosomal RNA genes (*12S rRNA* and *16S rRNA*), 22 transfer RNA (tRNA) genes, 13 protein-coding genes (PCGs), and one lengthy non-coding control region (CR). The mitogenome is a double-stranded circular molecule spanning a length of approximately 15–20 kb in animals [1]. Even though it originates from the first organelle captured by eukaryotes at the time of ancient competition [2], the mitogenome has several features that distinguish it from the nuclear genome, including a relatively fast evolutionary rate, matrilineal inheritance, conserved gene composition and arrangement, and few genetic recombination. Due to these features, the mitogenome has been universally used to study microevolution, population structure, molecular divergence time, phylogeography, and phylogenetics [3]. Owing to the rapid development of sequencing technology since the completion of the Human Genome Project in the 1990s, the next-generation sequencing (NGS) along with whole genome shotgun (WGS) approach is now widely used in mitogenome sequencing for various organisms (e.g., insects [4], birds [5], fishes [6], and frogs [7]).

The frog family Rhacophoridae is the fifth-largest known taxon of anurans, with a total of 450 species currently reported [8]. They are also known as “Old World tree frogs,” as their linage originated in mainland Asia (East/Southeast Asia) and subsequently radiated to most of the “Old World” region (specifically, Sub-Saharan Africa and southern and southeastern Asia up to Philippines and Japan) [8,9]. Rhacophoridae have evolved multiple inimitable reproductive modes to adapt to the transition from aquatic to arboreal habitats after the Cretaceous–Paleogene (K-Pg) mass extinction [9,10,11], but there are only seven rhacophorid mitogenomes publicly available, which has hampered the exploration of the evolutionary history of this unique taxon until now. Furthermore, the genus *Polypedates*, a subgroup of the family Rhacophoridae mainly distributed in Southeast Asia, has a controversial taxonomic status due to its indistinguishable interspecific morphological characteristics in addition to the fact that its early determination was based on only a few mitochondrial gene fragments [12,13,14]. However, no studies on *Polypedates* have examined the complete mitogenome for phylogenetic purposes. Moreover, whole mitochondrial data were available only for two of the four species found in China [15].

Overall, frog mitogenomes are fast-evolving. The mitochondrial genes of the Archaeobatrachia suborder retain the canonical vertebrate arrangement, whereas many neobatrachian frogs have been split into multiple novel derived orders with duplicated CR copies (e.g., Dicroglossidae [16,17], Mantellidae [18,19], and Rhacophoridae [20,21,22,23,24]). This duplication pattern seems to be phylogenetically conserved and is thought to result from either independent evolution [25,26] or concerted evolution [27,28,29]. This inherently leads to two questions: (1) which pattern led to the duplicated CRs that widely occur in rhacophorid tree frogs? and (2) how have paralogous CRs maintained such a high level of similarity?

Compared to gene rearrangements, mitochondrial gene loss is rare, especially for protein genes. Two extant complete mitogenomes of *Polypedates* tree frogs have been observed to have their *ATP8* genes become non-coding regions (NCRs) with distinct lengths [21,22]. In addition, the absence of the *ND5* gene in *P. megacephalus* has also been reported [30]. However, this gene loss event is questionable [18], as it was recently confirmed to be a mistake due to an assembling error caused by an unsuitable sequence strategy [22], the *ND5* gene being instead located in the middle of two CRs with very high sequence similarity. Nevertheless, the specific evolutionary process of *ATP8* gene loss of function (LoF) and the subsequent formation of NCRs with different lengths in *Polypedates* tree frogs has not been thoroughly investigated.

To address this knowledge gap, we sequenced and characterized the complete mitogenome of two *Polypedates* tree frogs, *P. impresus* and *P. mutus*. Based on comparative mitogenomic analysis of nine rhacophorids and phylogenetic analyses of 178 anurans, we (1) elucidated the mitogenomic features of *P. impresus* and *P. mutus*; (2) described the similarities and differences among the nine rhacophorid mitogenomes in detail; (3) inferred the evolutionary pathway and mechanisms leading to the formation of the variable NCR that replaced the original *ATP8* gene in the *Polypedates* lineage; (4) discussed the evolutionary pattern of CR duplication in rhacophorids and the maintenance process underlying the high sequence similarity between paralogous CRs; and (5) reconstructed the phylogenetic relationships among 178 anuran taxa based on sequence concatenation of 13 protein-coding genes (PCGs) and two rRNA genes.

## 2. Materials and Methods

### 2.1. Sample Collection and DNA Extraction

*Polypedates impresus* and *Polypedates mutus* were captured in Luchun County, Yunnan Province, and in Bawangling, Hainan Province, China. Tissue samples of each species were collected from a single individual. Tissue collection was approved by the Committee of the Ethics on Animal Care and Experiments at Sichuan Agricultural University (CEACE) (Permit Number: S2019202020) and was conducted according to the guidelines stated by CEACE. Collected tissues were immediately immersed in 99% ethanol and stored in a freezer at −20 °C. Total genomic DNA was extracted using the Ezup Columned Animal Genomic DNA Extraction Kit (Sangon Biotech, Shanghai, China) according to the manufacturer’s instructions. The extracts were prepared for downstream Sanger sequencing and NGS.

### 2.2. WGS Library Preparation

A portion of the DNA extracted from two individuals was sent to Personal Biotechnology (Shanghai, China) for WGS library construction. Samples were prepared using the TruSeq DNA Sample Preparation Kit (Illumina, San Diego, CA, USA), templated using the Template Prep Kit (Pacific Biosciences, Menlo Park, CA, USA), and then the sequencing libraries were generated. The sample concentration and purity were measured using the NanoDrop 2000 (Thermo Fisher Scientific, Waltham, MA, USA), and its integrity was measured using gel electrophoresis. A focused-ultrasonicator system (Covaris, Woburn, MA, USA) was used for DNA fragmentation. Then, two fragments of DNA with joints were selectively enriched and amplified. Sequencing on an Illumina MiSeq platform was performed with a single-stranded library as the template, a paired-read length of approximately 251 bp, and an average insert size of approximately 400 bp.

### 2.3. Genome Assembly and LA-PCR Amplification

The quality of the raw sequencing data (in FASTQ format) was evaluated using FastQC (http://www.bioinformatics.babraham.ac.uk/projects/fastqc/, accessed on 8 April 2020). Then, the raw data were cleaned, filtered, and assembled using the following steps. First, Adapter Removal v2 [31] was used to remove the adaptors that were incorporated in the Illumina reads. Second, the short reads were locally corrected using the k-mer strategy in the SOAPec v2.01 module of SOAP de novo2 (https://sourceforge.net/projects/soapdenovo2/, accessed on 8 April 2020). The sequencing reads were uploaded to NCBI Sequence Read Archive (SRA) (*P. impresus*, SRA accession number: PRJNA805066; *P. mutus*, SRA accession number: PRJNA805072). A5-miseq v20150522 [32] and SPAdes v3.9.0 [33] were used to produce high-quality data for de novo assembly, and then contigs and scaffolds were generated. The sequencing depths for the two species were: 44,673 (*P. impresus*) and 38,236 (*P. mutus*), respectively. Sequences with high sequencing depths were extracted from the splice sequences and compared to the NT library of the NCBI database using BLASTn (BLAST v2.2.31+) to select the mitochondrial sequences for each splice result. The mitochondrial splicing results obtained from the different software were then input to MUMMER v3.1 [34] for co-linear analysis to determine the contig positions and fill contig gaps. The results were corrected using Pilon v1.18 [35] to obtain the final mitochondrial sequence.

To fill the gaps in non-coding sequences, we employed long and accurate PCR (LA-PCR). Three sets of primers (Table 1) were used to amplify the F2 and F3 fragments of the mitogenome, as well as to amplify the F1 fragment for verification of the authenticity of the non-coding sequence between gene *tRNA^Lys^* and *ATP6.* The primers were developed in previous studies [20,36,37] or newly designed based on NGS sequences produced in this study, and they were checked for viability using primer design software such as Oligo 7 and Primer Premier 5.

LA-PCR was carried out in a 50-μL reaction cocktail containing 0.5 μL TaKaRa LA Taq (5U/μL), 5 μL 10 × LA Taq Buffer Ⅱ (Mg^2+^ Plus), 8 μL dNTP Mixture (2.5 mM each), 2 μL of each primer, 2 μL DNA template, and 30.5 μL ddH_2_O. The thermal profile was as follows: pre-denaturation at 94 °C for 3 min; 35 cycles of denaturation at 95 °C for 30 s, annealing at 48–55 °C for 45 s, extension at 72 °C for 4–5 min; and final extension at 72 °C for 10 min. Amplified products were screened on a 1.0% agarose gel, purified using SanPrep Column DNA Glue Recovery Reagent (Sangon Biotech, Shanghai, China), and cloned with pEASY-T5 Zero Cloning Kit (TransGen Biotech, Beijing, China). The cloned bacterial fluid was sent to BGI Genomics (Beijing, China) for Sanger dideoxy sequencing of the recombinant plasmid using M13 universal primers. Genomic sequences from NGS and LA-PCR were edited and assembled manually using SeqMan (Lasergene v7.1.0; DNAStar, Inc., Madison, WI, USA).

### 2.4. Mitogenome Annotation

Two assembled complete mitogenome sequences were uploaded to the MITOS web server (http://mitos2.bioinf.uni-leipzig.de/index.py, accessed on 10 February 2021) [38,39] for functional annotation. The tRNAscan-SE 2.0 web server (http://trna.ucsc.edu/tRNAscan-SE/, accessed on 10 February 2021) [40] and ARWEN (http://130.235.244.92/ARWEN/, accessed on 10 February 2021) [41] were then utilized to confirm the identities of the tRNA genes and their secondary structures. Protein genes were also re-identified using NCBI’s ORF Finder (https://www.ncbi.nlm.nih.gov/orffinder/, accessed on 10 February 2021) to ensure the correct reading frame. Seven known mitogenome sequences of rhacophorids [20,21,22,23,24,30,36,42] were used to manually correct the gene boundaries by aligning the orthologous genes using MEGA-X (https://megasoftware.net/ accessed on 10 February 2021) [43]. The putative origins of replication on the light strand (O_L_) sequences were identified using the MITOS web server, and then the O_L_ sequences and the flanking sequences were uploaded to Mfold (http://unafold.rna.albany.edu/?q=mfold, accessed on 10 February 2021) [44] and Forna (force-directed RNA; http://rna.tbi.univie.ac.at/forna/, accessed on 10 February 2021) [45] to determine the secondary structures. Conserved motifs of the control region (i.e., TAS: termination-associated sequences; CSB: conserved sequence blocks) were identified through comparison against the reference sequences from phylogenetically close taxa. Tandem repeats of the control region were detected using Tandem Repeats Finder v4.09 (https://tandem.bu.edu/trf/trf.html, accessed on 10 February 2021) [46] with a consensus match threshold of >90%. The copy numbers of the repeats were rounded off. The circular mitogenome map was generated using the CGView server (http://cgview.ca/, accessed on 10 February 2021) [47]. A + T content and relative synonymous codon usage (RSCU) were calculated using MEGA-X software, and AT skew and GC skew were calculated using the following formulas: AT skew = (A − T)/(A + T) and GC skew = (G − C)/(G + C) [48]. All the figures used in this study were generated using the default parameters of each software or online web server, and they were manually embellished using Adobe illustrator 2020 (Adobe Inc., San Jose, CA, USA).

### 2.5. Phylogenetic Analyses

To ensure the accuracy and extensiveness of the phylogenetic analyses, we downloaded the mitogenomes of all 178 anuran species that have been characterized to date (28 October 2021) from the NCBI reference sequence (RefSeq) database [49], as well as one recently determined Rhacophoridae mitogenome [24] (*Zhangixalus arboreus*, GenBank ID: LC565708) that is not archived yet and our two new rhacophorid mitogenomes (*P. impresus*, GenBank ID: MT869008; *P. mutus*, GenBank ID: MT869009). Four non-anuran vertebrates (*Aptenodytes forsteri*, *A. patagonicus*, *Alligator sinensis,* and *A. mississippiensis*) served as outgroups to root the phylogenetic tree. Thus, the final combined mitogenome set contained 185 species from 30 families. The list of species used in the phylogenetic analyses can be found in Table S1.

All phylogenetic streamlined analyses were performed using the PhyloSuite v1.2.2 platform [50], by which 13 protein-coding genes and two rRNA genes were extracted from the 185 species. Then, the 15 extracted gene sets were aligned in batches using MAFFT v7.313 [51] with “auto” strategy and codon/normal alignment mode. The aligned gene sets were further refined using the codon-aware program MACSE v2.03 [52], which preserves reading frames and allows the incorporation of sequencing errors or sequences with frameshifts. Ambiguously aligned fragments from the 15 gene sets were subsequently removed using Gblocks v0.91b [53]. Finally, the 15 processed gene sets were concatenated into a single aligned mitogenomic dataset of 12,858 bp in length. This assembled dataset was used for downstream Bayesian inference (BI) and maximum likelihood (ML) phylogenetic analyses. The best-fit partitioning scheme and nucleotide substitution models for the dataset, which had 41 predefined partitions (13 PCGs × 3 codon positions + 2 rRNA genes = 41 partitions), were determined using PartitionFinder v2.1.1 [54] and the greedy algorithm [55] with the AICc criterion and linked branch lengths. The selected best-fit partitioning schemes and models for both the BI and ML phylogenetic analyses are listed in Table S2. BI phylogenies were inferred using MrBayes v3.2.6 [56] with two independent runs and four independent Markov chain Monte Carlo chains running for 10,000,000 generations. Sampling was performed every 1000 generations, and the initial 25% of the sampled data were discarded as burn-in data. ML phylogenies were inferred using IQ-TREE [57] under the edge-linked partition model for 1000 standard bootstraps, as well as the Shimodaira-Hasegawa-like approximate likelihood-ratio test [58]. The resulting phylogenetic trees from both BI and ML analyses were pre-edited using the iTOL website [59] and manually embellished using Adobe illustrator 2020 (Adobe Inc., San Jose, CA, USA).

To elucidate the evolutionary patterns between duplicated CRs of rhacophorids, we selected nine anurans from the NCBI RefSeq database that have known additional CRs, seven rhacophorids with duplicated CRs, and an outgroup consisting of *Bombina bombina*, which has only a single CR. Tandem repeats within CR sequences were identified using Tandem Repeats Finder v4.09 with the default parameters. The conserved CR sequences were obtained after detected tandem repeats were removed, and then the sequences were aligned using MAFFT v7.313 with the “auto” strategy and normal alignment mode. The best-fit nucleotide substitution models “TVM+I+G” were determined using PartitionFinder v2.1.1. Finally, the downstream BI and ML phylogenies were analyzed as described above.

## 3. Results

### 3.1. Mitogenome Organization and Nucleotide Composition

**Figure 1:**
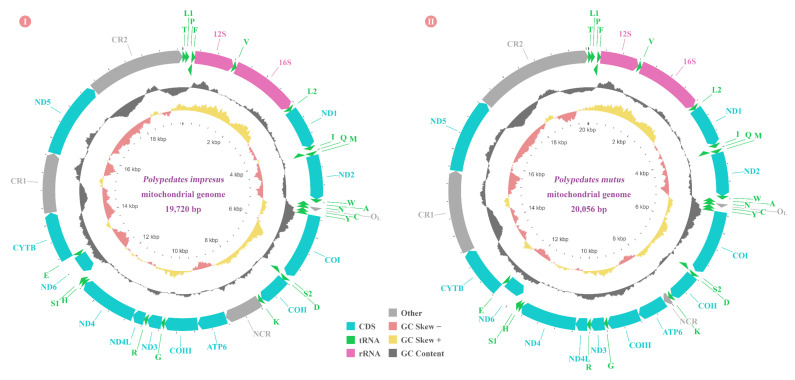
Mitogenome map of *Polypedates impresus* (**Ⅰ**) and *Polypedates* *mutus* (**Ⅱ**). Genes encoded on the heavy strand (H-strand) are plotted on the outer circle with clockwise arrows, and genes encoded on the light strand (L-strand) are plotted on the inner circle with counterclockwise arrows. Arrows represent the transcription orientation. Abbreviations: *ATP6*, adenosine triphosphate (ATP) synthase subunits 6; *COⅠ***–***Ⅲ*, cytochrome c oxidase subunits 1**–**3; *CYTB*, cytochrome b; *ND1***–***6* and *ND4L*, nicotinamide adenine dinucleotide hydrogen (*NADH*) dehydrogenase subunits 1**–**6 and 4L; *12S rRNA* and *16S rRNA*, small and large ribosomal RNA (rRNA) subunits; O_L_, origin of replication on the L-strand; NCR, noncoding region; CR, control region. Transfer RNA (tRNA) genes are indicated with their one-letter amino acid code of the corresponding amino acid. The length of features was drawn to match their actual nucleotide proportion in the mitogenome.

The complete mitogenomes of *P. impresus* and *P. mutus* were 19,720 bp and 20,056 bp in length, respectively, with a typical circular double-stranded configuration. The mitogenome of each frog species contained 12 PCGs with an absence of the *ATP8* gene but the presence of 22 tRNA genes, two rRNA genes (*12S rRNA* and *16S rRNA*), two CRs, one O_L_, and one NCR. This unique mitogenome composition was identical to that known for two other *Polypedates* frogs [21,22,30]. Eight tRNA genes (*tRNA^Pro^*, *tRNA^Gln^*, *tRNA^Ala^*, *tRNA^Asn^*, *tRNA^Cys^*, *tRNA^Tyr^*, *tRNA^Ser(UCN)^*, and *tRNA^Glu^*), and one PCG (*ND6*) were encoded on the light strand (L-strand), while the remaining 14 tRNAs and 11 PCGs were encoded on the heavy strand (H-strand) (Figure 1; Table S3). This H&L-strand gene distribution is congruent with all seven previously published rhacophorid mitogenomes [20,21,22,23,24,36,42]. Similar to the mitogenome of two other *Polypedates* frogs (*P. braueri* and *P. megacephalus*) [21,22,30], there was an NCR situated between gene *tRNA^Lys^* and *ATP6* that is thought to be the original position of the absent *ATP8* gene [22,30]. This NCR was 869 bp in *P. impresus*, but was only 149 bp in *P. mutus*.

Based on the nucleotide composition analysis rendered by MEGA-X, the nucleotide composition of the two complete mitogenomes was as follows: *P. impresus*: A: 30.2%, T: 30.8%, C: 14.7%, G: 24.3%; *P. mutus*: A: 30.5%, T: 30.4%, C: 14.5%, G: 24.6%. Both frogs showed distinct AT-bias, with an A+T content of 61.0% in *P. impresus* and 60.9% in *P. mutus*, which is in line with the other seven rhacophorids used in our study (Table 2). Furthermore, this genome-wide bias toward A and T also occurred in other individual PCGs and RNA genes (Table S4), and this bias was congruent with the mitogenomes of all seven previously reported rhacophorids, which ranged from a high of 64.5% (*Zhangixalus arboreus*) to a low of 60.4% (*Buergeria buergeri*) (Table S4).

The whole genome AT skew of *P. impresus* was slightly negative (−0.010), showing a higher content of A than T, whereas *P. mutus* had a positive mitogenomic AT skew value of 0.002. Both species showed a negative mitogenomic GC skew value (−0.246 for *P. impresus* and −0.258 for *P. mutus*), indicating a higher occurrence of C than G. This negative GC skew value was identical to that of the seven other rhacophorids (Table 2). As for the nucleotide composition analysis of individual genes, we found that twelve out of fifteen genes (*12S rRNA*, *16S rRNA*, *ND1*, *COⅠ*, *ATP6*, *COⅡ*, *COⅢ*, *ND3*, *ND4L*, *ND4*, *ND6*, and *CYTB*) in nine rhacophorids shared the same AT/GC skew polarity values, as shown in Table S4. The AT skew of four genes (*12S rRNA*, *16S rRNA*, *COⅡ*, and *ND6*) in the nine rhacophorids was positive, while the other eight genes (*ND1*, *COⅠ*, *ATP6*, *COⅢ*, *ND3*, *ND4L*, *ND4*, and *CYTB*) had a negative AT skew. The GC skew of all fifteen genes (two RNA genes plus thirteen PCGs, except the absent *ATP8* gene in *Polypedates* frogs) was negative in nine rhacophorids, with a higher content of C than G. Dissimilarity in the skews of the remaining three genes (*ND2*, *ND5*, *ATP8*) for the nine rhacophorids can be found in Table S4.

A total of 28 bp and 29 bp in 10 and 11 intergenic spacers were found in *P. impresus* and *P. mutus*, respectively (Table S3). The longest spacers were 5 bp and were located between gene *tRNA^Leu(CUN)^* and *tRNA^Pro^* as well as between gene *COⅡ* and *tRNA^Lys^*, which are the same positions known in two other *Polypedates* frogs [21,22,30]. The other spacers in *P. impresus* were found between gene *tRNA^Tyr^* and *COⅠ* (4 bp), *tRNA^Glu^* and *CYTB* (4 bp), *tRNA^Leu(UUR)^* and *ND1* (3 bp), *16S rRNA* and *tRNA^Leu(UUR)^* (2 bp), *tRNA^Ser(AGY)^* and *ND6* (2 bp), *tRNA^Pro^* and *tRNA^Phe^* (1 bp), *tRNA^Ala^* and *tRNA^Asn^* (1 bp), and *tRNA^Ser(UCN)^* and *tRNA^Asp^* (1 bp). *P. mutus* had the same spacers as *P. impresus* but had an additional spacer between gene *tRNA^Arg^* and *ND4L* (1 bp). Seven overlapping sites were observed, ranging from 1 to 13 bp (29 bp in total) with the same locations found in *P. impresus* and *P. mutus* (Table S3). The longest overlaps were located between gene *tRNA^Ser(UCN)^* and *tRNA^Asp^,* with 13 shared nucleotides. The other overlaps in *P. impresus* and *P. mutus* were between gene *ND4L* and *ND4* (7 bp), O_L_ and *tRNA^Cys^* (3 bp), *tRNA^Phe^* and *12S rRNA* (2 bp), *ND2* and *tRNA^Trp^* (2 bp), *tRNA^Ile^* and *tRNA^Gln^* (1 bp), and *tRNA^Gln^* and *tRNA^Met^* (1 bp). This unique distribution as well as the size and overlap of the intergenic spacers are almost identical to those of two other *Polypedates* frogs [21,22,30].

### 3.2. Protein-Coding Genes and Codon Usage

The total PCG length of *P. impresus* was 11,124 bp and it encoded 3706 amino acids, accounting for 56.41% of the whole genome. The total PCG length of *P. mutus* was 11,118 bp and it encoded 3704 amino acids, accounting for 55.43% of the whole genome. Comparing the nine rhacophorids, differences in the A+T content between the species did not exceed 3.3% (highest 62.3% in *Z. omeimontis* to lowest 59.0% in *B. buergeri*) and they all displayed significant bias toward A and T (Table 2). Among the nine rhacophorids, the AT skew across all PCGs was negative and ranged from −0.105 (*P. braueri*) to −0.048 (*Z. arboreus*). The GC skew in the PCGs was also negative, ranging from −0.286 (*Z. arboreus*) to −0.197 (*P. braueri*) (Table 2). Among all 12 individual PCGs of the nine Rhacophoridae species (except for the absent *ATP8* gene in four *Polypedates* species), the highest A+T content was in the *ND2* gene, with an average of 63.3% and a range from 60.6% (*Z. schlegelii*) to 65.1% (*Z. omeimontis*). The lowest A+T content was in the *CYTB* gene, with an average of 57.3% and a range from 53.4% (*B. buergeri*) to 60.1% (*P. braueri*) (Table S4).

Nearly all of the start codons of *P. impresus* and *P. mutus* were the conventional start codon ATN (N stands for A, T, and G), except for *ND4* gene in *P. mutus*, which was initiated with GTG (Table S3). For both *P. impresus* and *P. mutus*, truncated termination codon T-- was the most prevalent stop codon, as it was present in gene *COⅢ*, *ND1*, *ND3*, *ND4*, *ATP6*, and *CYTB*. In contrast, gene *COⅡ*, *ND4L*, and *ND5* ended with TAA; gene *COⅠ* and *ND6* ended with AGG; and only one PCG (*ND2*) ended with TAG (Table S3). As shown in Table S5, compared with four *Zhangixalus* species and one *Buergeria* species, the start and stop codons in all four *Polypedates* species were more conservative and shared nearly the same set of start/stop codons; however, exceptions included the *ND4* gene in *P. impresus* and *P. megacephalus*, which was initiated with the GTG codon, whereas *P. mutus* and *P. braueri* were initiated with ATG, and the *ND2* gene in *P. impresus* and *P. mutus*, which was terminated by TAG, whereas *P. megacephalus* and *P. braueri* were terminated by the incomplete termination codon T--. We also found that seven PCGs in the nine rhacophorids used the same set of start/stop codons. ATG was a common start codon for gene *CYTB*, *ND4L*, and *ND6* in all nine rhacophorids; the incomplete stop codon T-- was present in gene *ND1*, *ND3*, and *ND4*; and AGG was the common stop codon for *COⅠ* gene in all nine Rhacophoridae species (Table S5).

**Figure 2:**
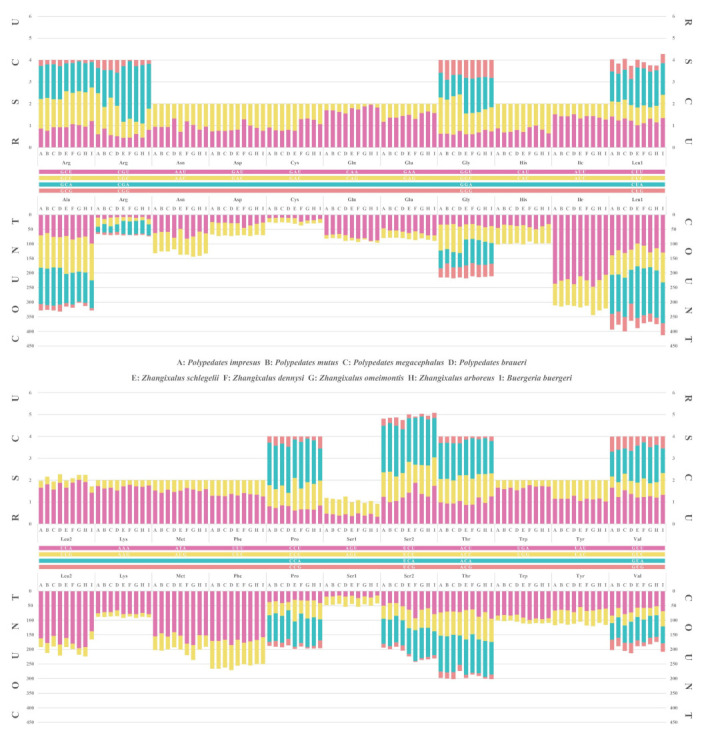
Amino acid and codon usage in the protein-coding genes (PCGs) of nine rhacophorid mitogenomes. The *X*-axis represents 22 codon families. The *Y*-axis on the upper *X*-axis represents the relative synonymous codon usage (RSCU), while the lower represents the number of different codons employed for each amino acid. Detailed numerical information can be accessed in Table S6. (A: *Polypedates impresus*; B: *Polypedates mutus*; C: *Polypedates megacephalus*; D: *Polypedates braueri*; E: *Zhangixalus schlegelii*; F: *Zhangixalus dennysi*; G: *Zhangixalus omeimontis*; H: *Zhangixalus arboreus*; I*: Buergeria buergeri*).

Comparative codon usage analysis for the nine rhacophorids showed that the codon usage patterns were conserved. All possible synonymous codons of the 22 amino acids are presented in Table S6. According to Figure 2, the most frequently encoded amino acid was leucine, with six codons ranging from 15.96% (*Z. arboreus* with 599 cases) to 15.39% (*B. buergeri* with 579 cases), while cysteine was the rarest, ranging from 0.70% (*P. impresus* with 26 cases) to 0.83% (*Z. schlegelii* with 31 cases). Further RSCU analysis showed that the codon AUU, which encodes isoleucine, was the most frequently used codon in the nine rhacophorids, accounting for 5.47% (*B. buergeri* with 206 cases) to 6.51% (*Z. omeimontis* with 247 cases). The exact proportion of each codon set can be found in Table S6. The highest RSCU values among the nine rhacophorids were much more complex. Three out of the four *Polypedates* species had the highest RSCU value for UCA, which encodes serine (*P. impresus*, 2.13; *P. mutus*, 2.22; and *P. megacephalus*, 2.32), whereas *P. braueri* had the highest RSCU value for CCA (encoding proline) at 2.11. As for the four *Zhangixalus* species, their highest RSCU values were all for CGA, which encodes arginine, with 2.67 for *Z. arboreus*, 2.63 for *Z. dennysi*, and 2.54 for both *Z. schlegelii* and *Z. omeimontis*. The highest RSCU value in *B. buergeri* was for CAA, which encodes glutamine, with a value of 1.83 (Table S6). Furthermore, RSCU analysis also indicated that codons including A or T in the third codon position were always overused compared to other synonymous codons. Except for *P. megacephalus*, the third codon positions that had A or T (range: 59.8% of *P. braueri* to 70.8% of *Z. dennysi*) in the remaining eight rhacophorids were always in a higher proportion than the second (57.2–60.5%) and the first (52.7–60.8%) codon positions. In contrast, in *P. megacephalus,* the AT content of the third codon position (59.3%) was slightly lower than that of the second codon position (59.6%).

### 3.3. Transfer and Ribosomal RNA Genes

All 22 tRNA genes were identified in *P. impresus* and *P. mutus*, and a total of 1543 bp and 1535 bp were occupied in their respective mitogenomes. The size of each individual tRNA gene in the two frogs was between 65 and 74 bp. Analysis of the concatenated sequence of the 22 tRNA genes showed that both AT skew and GC skew were positive and consistent with the other seven rhacophorids used in this study, suggesting that rhacophorid tRNA genes with AG are more favored than those with TC (Table 2). Furthermore, we found that the AT content of the rhacophorids ranged from 56.3% to 59.8%, suggesting a slight bias toward AT compared to GC (Table 2). Similar to the two published *Polypedates* mitogenomes as well as most vertebrate mitogenomes, 14 tRNAs were encoded by the H-strand, and the remaining eight tRNAs were encoded by the L-strand. Four tRNA genes were arranged into *tRNA^Thr^*/*tRNA^Leu (CUN)^*/*tRNA^Pro^*/*tRNA^Phe^*, forming a “TLPF” tRNA gene cluster (Figure 1). Similar to most vertebrates, five tRNA genes (*tRNA^Trp^*/*tRNA^Ala^*/*tRNA^ASN^*/*tRNA^Cys^*/*tRNA^Tyr^*) formed a “WANCY” cluster (Figure 1) [22].

**Figure 3:**
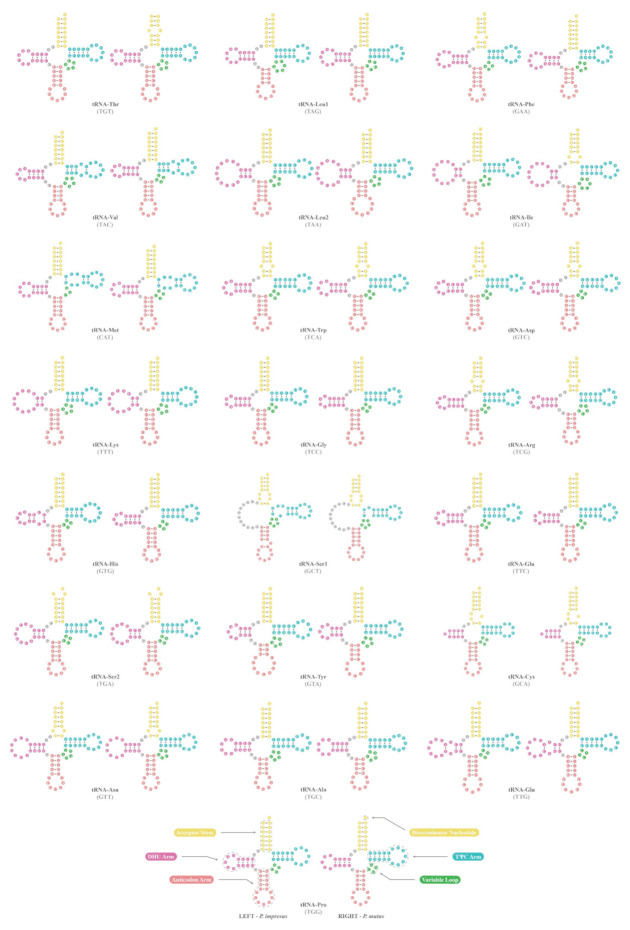
Putative secondary structures for 22 tRNA genes in the mitogenome of *Polypedates impresus* (left) and *Polypedates mutus* (right).

All 22 tRNAs could be folded into a typical clover-leaf structure, with the exception of gene *tRNA^Ser(AGN)^* and *tRNA^Cys^* (Figure 3). *tRNA^Ser(AGN^^)^* could not form an entire DHU arm, as it only formed a loop structure, and *tRNA^Cys^* did not have a complete DHU arm but rather had a bare stem structure. Apart from the genes *tRNA^Leu(CUN)^*, *tRNA^Leu(UUR)^*, and *tRNA^Tyr^* in both *Polypedates* species, *tRNA^Ile^* from *P. impresus* and *tRNA^Lys^* from *P. mutus* did not contain any mismatched pairs (Figure 3). A total of 33 and 37 non-Watson-Crick base pairs were found in *P. impresus* and *P. mutus*, respectively. Most of the mismatched pairs were G-U pairs (19/33 pairs in *P. impresus* and 23/37 pairs in *P. mutus*).

Both the *12S rRNA* gene and *16S rRNA* gene of *P. impresus* and *P. mutus* were encoded on the H-strand, located between gene *tRNA^Phe^* and *tRNA^Leu(UUR)^*, and separated by *tRNA^Val^* (Figure 1). This three-gene cluster is common to most vertebrates. The length of the *16S rRNA* gene was 1537 bp for *P. impresus* and *P. mutus*, and the size of *12S rRNA* gene was 930 bp for *P. impresus* and 931 bp for *P. mutus* (Table S3). As shown in Table 2 and Table S4, both rRNA genes presented a high A+T preference of 59.0% for *P. impresus* and 59.9% for *P. mutus*, which was in line with the other seven rhacophorids [20,21,22,23,24,30,36,42]. Furthermore, the comparative analysis indicated that all nine Rhacophoridae species possessed a positive AT skew and a negative GC skew for both the *12S rRNA* gene and *16S rRNA* gene (Table S4).

### 3.4. O_L_ of Nine Rhacophorids

**Figure 4:**
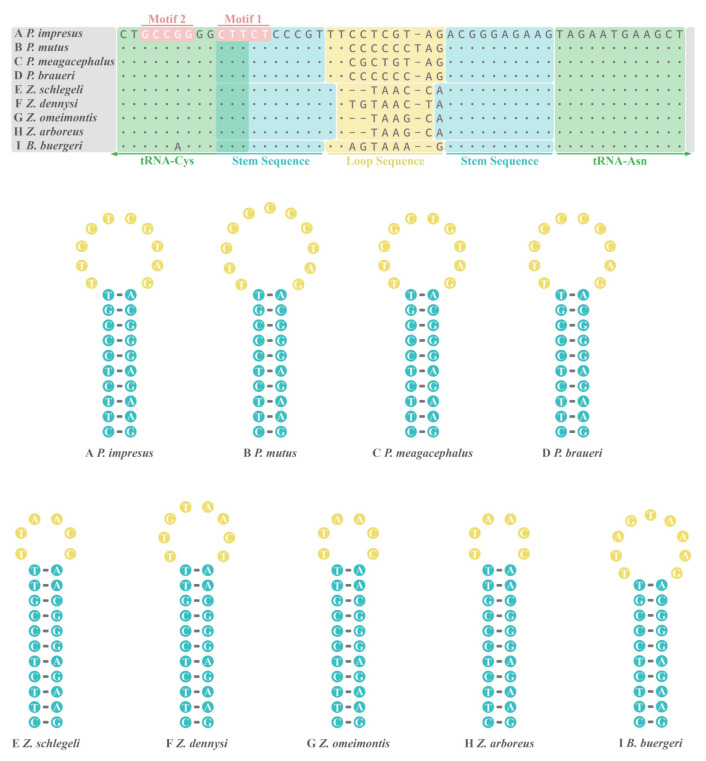
Putative secondary structures and sequence alignments of origin of replication on the light strand (O_L_) in the mitogenome of nine rhacophorids.

The putative lengths of O_L_ in *P. impresus* and *P. mutus* were 30 bp and 31 bp, respectively, and both were located within a “WANCY” tRNA gene cluster and between genes *tRNA^Asn^* and *tRNA^Cys^* (Figure 1). Compared with all nine currently available rhacophorid mitogenomes, we found that indels almost occurred in the loop region (Figure 4). All nine frogs shared the same 5′-CTTCTCCCGT-3′ stem sequence, except for four *Zhangixalus* species that had an extra A-T base pair in the stem. The O_L_ of all nine rhacophorids shared a 3-bp overlap with upstream *tRNA^Cys^* gene while having no spacer nor overlap with downstream *tRNA^Asn^*. The O_L_ of four *Polypedates* species and one *Buergeria* species were encoded on the H-strand, whereas those of four *Zhangixalus* species were encoded on the L-strand, with three of them (*Z. schlegelii*, *Z. omeimontis*, and *Z. arboreus*) sharing the same O_L_ structure (Figure 4).

### 3.5. CR of Nine Rhacophorids

**Figure 5:**
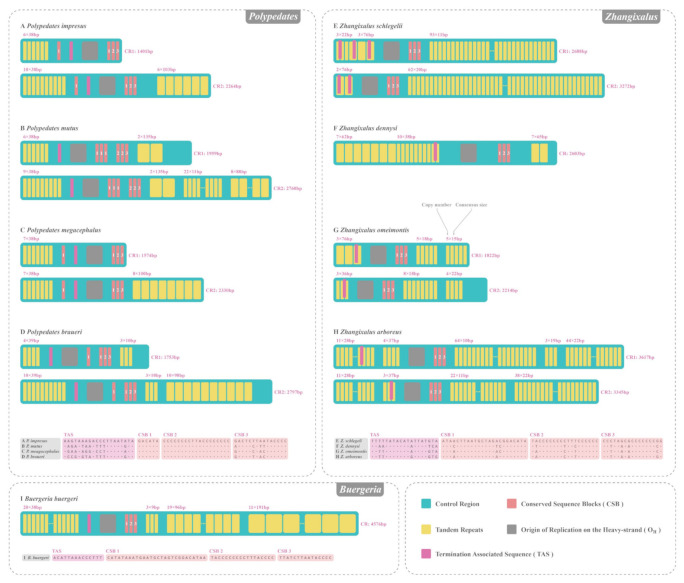
Mitogenome control region (CR) structure comparison and conservative sequence alignments of nine rhacophorids. Structures of the CRs and putative termination associated sequences (TAS), conserved sequence blocks (CSB), and tandem repeats are plotted for each species.

Both *P. impresus* and *P. mutus* sequenced in this study contained two CRs at the same positions, and these were labeled CR1 (between gene *CYTB* and *ND5*) and CR2 (between *ND5* gene and the “TLPF” tRNA gene cluster) (Figure 1). The lengths of CR1 and CR2 in *P. impresus* were 1401 and 2264 bp, respectively, while those in *P. mutus* were 1959 and 2760 bp, respectively (Figure 5). The nucleotide composition of CR1 in *P. impresus* was 32.8% A, 31.5% T, 23.8% C, and 11.9% G; and that of CR2 was 34.0% A, 35.3% T, 19.8% C, and 10.9% G, yielding higher AT content (64.3% and 69.3% for CR1 and CR2, respectively) in the CRs than in the whole genome (61.0%). The nucleotide composition of CR1 in *P. mutus* was 33.4% A, 32.0% T, 23.4% C, and 11.2% G; and that of CR2 was 33.5% A, 33.5% T, 22.1% C, and 10.8% G, thus also showing higher AT content (65.4% and 67.0% for CR1 and CR2, respectively) in the CRs than in the whole genome (60.9%). Both *Polypedates* examined here had nearly identical sequences at the 5′-end of the duplicated CRs (91.1% similarity with 133 substitutions in 1496 alignable sites of *P. impresus*, 99.7% similarity with only five substitutions in 1690 alignable sites of *P. mutus*). The high sequence similarity of the 5′-end of homologous CRs was also observed in five other rhacophorids with additional CRs (detailed numerical information for *P. megacephalus*, *P. braueri Z. schlegelii*, *Z. omeimontis,* and *Z. arboreus* is shown in Table 3). Duplicated CRs located on the flank of the *ND5* gene were found in all nine Rhacophoridae species, except for *Z. dennysi* and *B. buergeri*, in which only a single CR was observed between gene *CYTB* and *ND5* (Figure 5).

**Figure 6:**
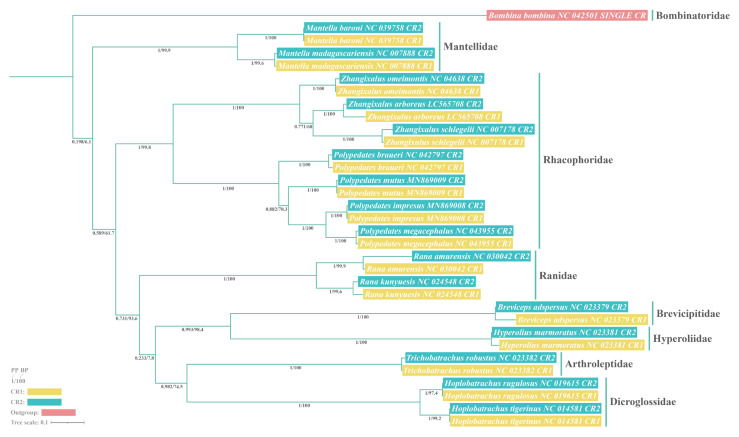
Phylogenetic tree based on control region (CR) sequences of 16 anurans with duplicated CRs. *Bombina bombina* with only a single CR was selected as the outgroup. Both maximum likelihood (ML) and Bayesian inference (BI) analyses yielded identical topologies with comparable branch support. Paralogous CRs were always clustered together with strong node support, indicating that the duplicated CRs within those individuals were evolved in concert.

There are two distinct evolutionary explanations for this novel phenomenon of duplicated CRs occurring within individual mitogenomes: independent evolution [25,26] and concerted evolution [27,28,29]. To elucidate the exact evolutionary patterns of all the rhacophorids with repeated CRs involved in this study, we additionally considered nine anurans with different CRs from the NCBI RefSeq database, the seven rhacophorids with duplicated CRs, and *Bombina bombina* with only a single CR (as outgroup). Phylogenetic analyses of the conserved sequences obtained after removing the tandem repeats from the CRs of the 17 anuran species revealed the same topology in both BI and ML (Figure 6).

As shown in Figure 6, paralogous CRs (i.e., CR1 and CR2 from common individuals) were always more closely related than orthologous CRs (i.e., all CR1s from every species and all CR2s from every species), with strong support for the clustering of the terminal nodes of CR1 and CR2 within common individuals. This phylogenetic tree for the 17 frog species suggested that dual CRs within those individuals apparently evolved in concert. In contrast, independent evolution would have resulted in separate clusters of CR1s and CR2s from different species. This concerted evolution pattern of CRs was also clearly portrayed by the sequence similarity in Table 3, as the sequences similarity between the CRs of each species was as high as 91.1–99.9%. For a given CR pair, independent evolution will lead to differences in the CR sequences that may consequently result in degradation or deletion of one of the CRs [60,61]. However, concerted evolution can maintain a high degree of sequence similarity between the two CRs through homogenization.

### 3.6. Gene Rearrangements of Nine Rhacophorids

**Figure 7:**
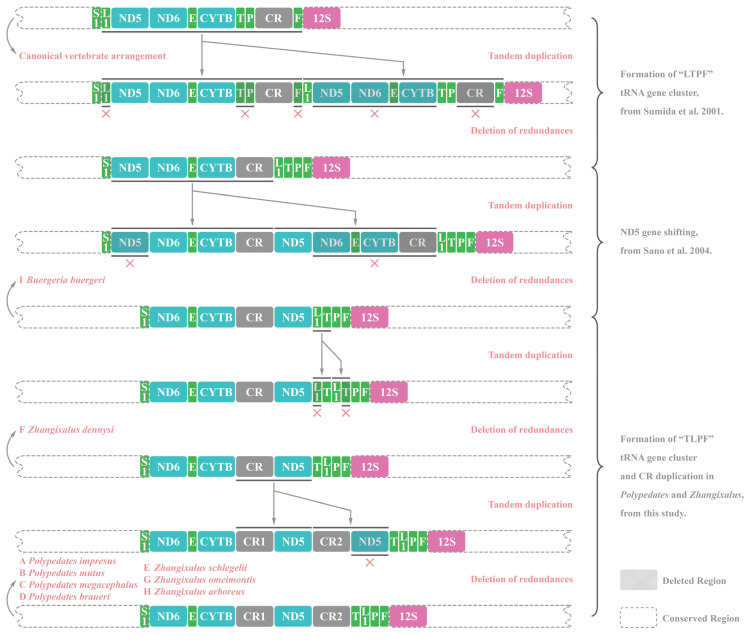
Diagram of the presumptive gene rearrangement pathway with tandem duplication and random loss (TDRL) processes. Step formation of “LTPF” tRNA gene cluster from Sumida et al. [62]. Step ND5 gene shifting from Sano et al. [36]. The length of features was not drawn to match their actual nucleotide proportion in the mitogenome.

Compared with the standard vertebrate gene arrangement (e.g., that of *Xenopus laevis*), two gene rearrangements occurred in *Polypedates*, *Zhangixalus,* and *Buergeria* frogs. The first rearrangement was the transferring of *ND5* gene from its original position between gene *ND4* and *ND6* to an area downstream of the CR. The second rearrangement was the shift in the position of gene *tRNA^Thr^*, *tRNA^Leu^*, *tRNA^Pro^*, and *tRNA^Phe^* to form a “TLPF” tRNA gene cluster located upstream of the *12S rRNA* gene. Previous studies have inferred the evolutionary pathway from the typical vertebrate condition with the tandem duplication and random loss (TDRL) model [20,30,36,62]. Therefore, based on the principle of parsimony, we made some complements to fit the existing gene order and phylogenetic relationships of the nine rhacophorids, as shown in Figure 7. Overall, all presumed gene duplications and subsequent deletion events were concentrated between gene *tRNA^Ser(AGY)^* (S1 in Figure 7) and *12S rRNA* (12S in Figure 7).

**Figure 8:**
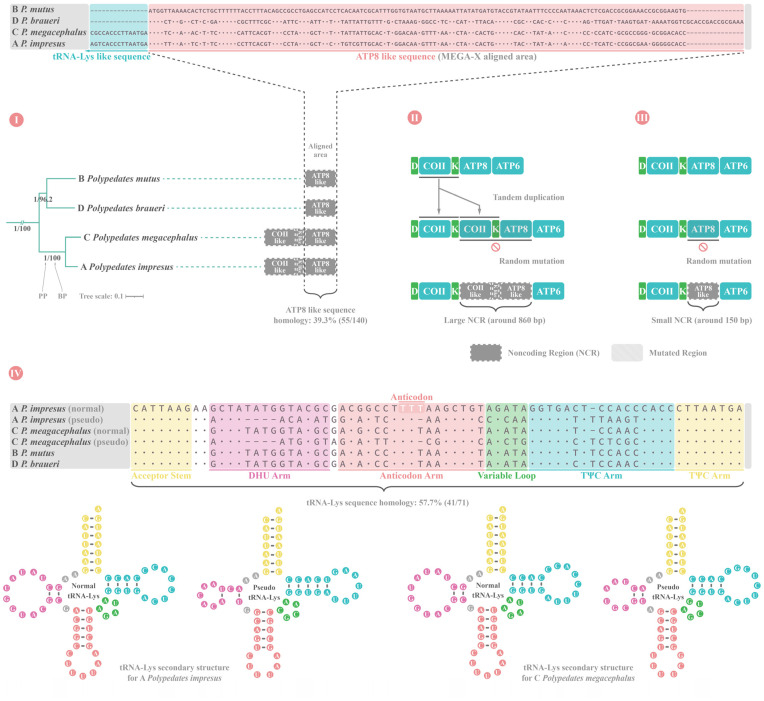
Diagram of the presumptive noncoding region (NCR) formation pathway and component analysis of four *Polypedates* frogs. (**Ⅰ**) Phylogenetic relationship for four *Polypedates* and their NCR sequence alignment; (**Ⅱ**) Putative NCR formation pathway for *Polypedates impresus* and *Polypedates megacephalus*; (**Ⅲ**) Putative NCR formation pathway for *Polypedates mutus* and *Polypedates braueri*; (**Ⅳ**) Gene *tRNA^Lys^* sequence alignment and secondary structure prediction for four *Polypedates*. The length of features was not drawn to match their actual nucleotide proportion in the mitogenome. (A: *Polypedates impresus*; B: *Polypedates mutus*; C: *Polypedates megacephalus*; D: *Polypedates braueri*).

It is worth noting that none of the four *Polypedates* species used in this study had the functional *ATP8* gene sequence, indicating that the newly sequenced *P. impresus* and *P. mutus* also lost their *ATP8* gene. A study by Zhang et al. [30] on *P. megacephalus* suggested that *ATP8* gene had become an NCR located between gene *tRNA^Lys^* and *ATP6* (Figure 8Ⅱ), we also found an NCR in the same position for *P. impresus*, *P. mutus,* and *P. braueri*. Interestingly, the NCR sizes differed somewhat between the four *Polypedates* frogs. *P. impresus* and *P. megacephalus* had larger NCRs of 860 and 853 bp, respectively, whereas *P. mutus* and *P. braueri* had smaller NCRs of 140 and 155 bp, respectively (Figure 8Ⅱ and Figure 8Ⅲ). We also found a putative *tRNA^Lys^* pseudogene in the large NCR sequence of *P. impresus* that may not function normally due to abnormal tRNA secondary structure, particularly an anticodon loop lacking a nucleotide that likely prevents it from correctly identifying the corresponding codon for lysine (Figure 8Ⅳ).

### 3.7. Phylogenetic Relationships

**Figure 9:**
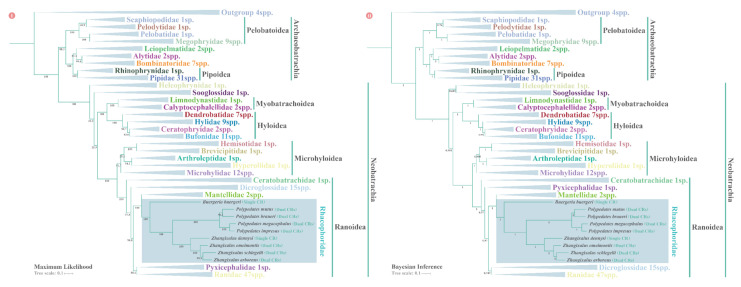
Phylogenetic tree for 185 species inferred from nucleotide sequences of 13 protein-coding genes (PCGs) and two rRNA genes of the mitogenome with maximum likelihood (ML) (**Ⅰ**) and Bayesian inference (BI) (**Ⅱ**) analyses. The numbers below the branches represent the bootstrap proportion (BP) and posterior probability (PP) values. All the families except Rhacophoridae are collapsed for concise presentation. Detailed topologies of species within each family can be accessed in Figures S1 and S2.

Both ML and BI phylogenetic analyses for 28 anuran families yielded overall identical topologies with comparable branch support, but they did differ in terms of the branching order and cluster relation of some nodes (Figure 9).

Two major clades were recovered robustly: the Archaeobatrachia suborder, which contains various primitive frogs and toads, and the Neobatrachia suborder, which accounts for more than 96% of all living anurans [63]. All species were clustered at the family level into branches with strong node support (Figures S1 and S2). The only controversy between the ML and BI analyses is concentrated in five families: Heleophrynidae, Sooglossidae, Dicroglossidae, Pyxicephalidae, and Ranidae. In the ML analysis, Heleophrynidae split before Sooglossidae, while these taxa were sister in the BI analysis. As for the family Dicroglossidae, Pyxicephalidae, and Ranidae, the ML analysis recovered (((Mantellidae + Rhacophoridae) + (Pyxicephalidae + Ranidae)) + Dicroglossidae) topology, whereas BI analysis demonstrated (((Mantellidae + Rhacophoridae) + (Dicroglossidae + Ranidae)) + Pyxicephalidae) arrangement. Both the ML and BI results had a modest branch support (BP: 57.8–90.8; PP: 0.949–0.97).

Within the target lineage Rhacophoridae, both ML and BI trees demonstrated the following arrangement: (*B. buergeri* + (((*P. mutus* + *P. braueri*) + (*P. megacephalus* + *P. impresus*)) + (*Z. dennysi* + (*Z. omeimontis* + (*Z. schlegeli* + *Z. arboreus*))))). This arrangement received strong support from both BI and ML analyses (BP: 96.2–100; PP: 1 for all nodes). And we also noticed that *B. buergeri* is located at the basal phylogenetic position of the family Rhacophoridae. As for four *Polypedates* species, the pairs *P. mutus* and *P. braueri*, and *P. megacephalus* and *P. impresus* formed separate decisive sister groups (BP: 96.2–100; PP: 1 for all nodes).

## 4. Discussion

### 4.1. Mitogenome Sructural Analyses of Nine Rhacophorids

All nine rhacophorid mitogenomes demonstrated genome-wide bias toward A and T, which ranged from a high of 64.5% (*Z. arboreus*) to a low of 60.4% (*B. buergeri*) (Table 2). A recent experiment has proposed a hypothesis for the existence of such a high AT content, which is that nucleotide biosynthesis requires a large amount of materials and energy, especially for transcribed sequences that are often amplified thousands of times more than the genomic sequences, so choosing a higher AT content for encoding can save limited intracellular resources and energy consumption during transcription [64,65]. The presence of multiple small intergenic spacers and overlaps between genes of two *Polypedates* frogs is indicative of the intense selective pressure for minimization in metazoan genomes [66]. Some invertebrates even reduce sequence length to streamline the structure of some tRNAs as a trade-off for genomic parsimony, as observed in snails [67] and spiders [68,69]. This asymmetry in nucleotide composition is often seen as an indicator of gene orientation and replication direction during gene replication and transcription [48,70].

In the codon analysis of two *Polypedates* frogs, truncated termination codon T-- was the most frequent stop codon, which was observed in genes *COⅢ*, *ND1*, *ND3*, *ND4*, *ATP6*, and *CYTB*. Multiple incomplete termination codons are common phenomena in metazoans [1,71]. These abnormal T-- termini will presumably be completed by transcription processes via post-transcriptional polyadenylation [72]. As compared with four *Zhangixalus* and one *Buergeria* species (Table S5), the start and stop codons in the entire *Polypedates* lineage were conserved. As for the RSCU analysis of two *Polypedates* frogs, the phenomenon of AT bias is particularly pronounced at the third codon position, was also reflected in the codon frequencies (Table S6), and has been previously reported for other vertebrates [73,74] and invertebrates [65,75,76,77].

With regard to the abnormal structures in the tRNA genes in *P. impresus* and *P. mutus* (Figure 3), the incomplete DHU arm structure of *tRNA^Ser(AGN)^* was pointed out as being a typical structure of metazoan mitogenomes in an early study [78]. However, the *tRNA^Cys^* did not have a complete DHU arm; instead, a unique stem structure was reported only in this study and two other *Polypedates* frogs [21,22]. Those incomplete clover-leaf structures might function normally through the structural compensation mechanism between the tRNA arms, and they should not affect transport functions [79]. Another type of abnormal structure was found when most of the mismatched pairs were G-U pairs in tRNA genes of *P. impresus* and *P. mutus*. Due to their comparable thermodynamic stability and isomorphism to canonical Watson-Crick base pairs (i.e., A-U(T) and G-C pairs), G-U pairs often replace canonical pairs in RNAs [80]. The G-U pair is probably repaired through a presumed RNA-dependent RNA polymerase [81].

As a conserved structure that can fold into a stable hairpin secondary structure with a loop and a stem in the mitogenome [82], O_L_ plays an essential role in identifying the initial DNA polymerase site and facilitating the accurate initiation of DNA synthesis [73,82,83]. By the results of O_L_ sequence alignment of nine rhacophorid, we found that indels almost occurred in the loop region (Figure 4). In a previous study has pointed out the high variation in the loop area did not reduce the level of DNA replication [83]. We also found that, expectedly, motif 1 (5′-CTTCT-3′) (Figure 4) occurred in all nine rhacophorids. This motif thought to be a strictly conserved sequence in all vertebrates, and it may facilitate locating the O_L_ [83]. Interestingly, motif 2 (5′-GCCGG-3′) at the 5′ base of the stem was thought to be concretive and necessary for the replication of the L-strand [83,84]. Motif 2 has been found in other vertebrates [73,84,85], and a variation of it (5′-GCCAG-3′ as opposed to 5′-GCCGG-3′) was found in *B. buergeri* and has coincidentally also been found in *Cricetulus* hamsters [86]. The most significant mutations in the O_L_ of vertebrates occur in birds, in which the entire O_L_ is degraded and deleted from the mitogenome [87,88,89].

### 4.2. CR Sructural Analyses of Nine Rhacophorids

Colossal and duplicated CRs are the chief factors responsible for the relatively large mitogenome size in some anurans [18,90,91]. For instance, *B. buergeri* has the largest known rhacophorid CR (a single CR that is 4576 bp long) [36], and *Breviceps adspersus* in the family Brevicipitidae has the largest CR of all known anurans (CR over 6.5 kbp), and this species also has the largest mitogenome size among known vertebrates (a total of 28,757 bp) [92]. The polymorphism of CR length is determined by tandem repeats with variable size and copy numbers [84,90,91], which led to variations in the mitogenome sizes of the nine rhacophorids involved in this study (Figure 5). Variations in the size of metazoan mitogenomes were determined by tandem repeats within the CR, which have also been confirmed in studies on the mitogenomes of fish, reptiles, birds, mammals, and insects [1]. As shown in Figure 5, tandem repeats were mainly distributed at both ends of the mitogenome. *B. buergeri* undoubtedly possessed the largest length of repeated units (over 4 kbp), while *Z. omeimontis* had the shortest (approximately 730 bp) among the nine rhacophorids. Further comparison of the tandem repeats within the same genus revealed that the variation between the CR internal structure of *Zhangixalus* was significant, but it was difficult to reveal any commonality among the four *Zhangixalus* species. The tandem repeat arrangement within *Polypedates* was much more conservative. In particular, the small repeats at the 5′-end of the CRs in *Polypedates* showed a similar pattern (multiple 38–39 bp copies at the 5′-end of the CRs in all four *Polypedates* were detected, with 28.9% [11/38] sequence similarity between the eight tandem repeats), and unexpectedly, the 5′-ends of CR1 in both *P. impresus* and *P. megacephalus* were exactly the same sequence (ATTTACCCCATCATACTATGTATAATAAGCATTAATTT), suggesting that the 5′-end repeats of *Polypedates* taxa might originate from a common ancestor. However, the nucleotide sequences and structural patterns of the 3′-end repeats differed among the nine rhacophorids. The considerable variation in CR structure in *Zhangixalus* compared to *Polypedates* is likely attributable to the fact that *Zhangixalus* was separated from the genus *Rhacophorus* (Kuhl and Van Hasselt, 1822) as a new genus just two years ago, and its intricate interspecific relationships still need further study [93].

In addition to the tandem repeats, several characteristics of the CRs, such as termination-associated sequences (TASs) and conserved sequence blocks (CSBs), were also identified in the *P. impresus* and *P. braueri* examined in this study, as well as seven other rhacophorids (Figure 5). TASs are thought to potentially bind to specific proteins to regulate mitogenome synthesis [94]. Among the nine rhacophorids, only one TAS was found in each CR of each species except for *Z. schlegelii*, which was observed to have duplicated TAS (three and two TASs were detected upstream of CR1 and 2, respectively). In addition, the TASs of *Zhangixalus* were embedded in tandem repeats upstream of the CRs, while *Polypedates* and *Buergeria* frogs were isolated from the tandem repeats (Figure 5). This insertion of TAS into tandem repeats has also been observed in some Bufonidae and Hylidae taxa, and TAS repeats within a single CR have also been found in these two lineages [90]. Although more species verification is required, we can hypothesize that the mutual position between TAS and tandem repeats of *Polypedates* and *Zhangixalus* may have a solid intrageneric correlation. Future definitions of the CR of these two genera may also exhibit the same pattern.

As another type of conserved motif within the CR, it has been suggested that multiple CSBs may be essential for the synthesis of D-loop DNA [95], which seems to be formed in the closed DNA by displacement synthesis of a short progeny strand, with a specific region of the mitogenome L-strand serving as a template [96]. Comparison of CSBs among nine rhacophorids revealed that four *Polypedates* had the most conserved CSB1 and 2 with completely identical sequences, while the CSBs of the remaining taxa were relatively less conserved, with a few base mutations detected (Figure 5). Unlike the TAS motif mentioned above, none of the CSBs in the nine rhacophorids were found to be inserted in the tandem repeats. This independent pattern of CSBs has also been reported in some Discoglossidae frogs [97]. In contrast to the relatively complex arrangement of CSBs in *Polypedates*, we found that the three CSBs (CSBs 1, 2, and 3) in *Zhangixalus* and *Buergeria* were always clinging to each other and had no additional copies. Combined with the phylogenetic relationships of the nine rhacophorids (Figure 9), this result suggests that the triplet CSB arrangement is an ancestral state that originated in *Buergeria* and is conserved in *Zhangixalus*, and that the *Polypedates* lineage with reorganized CSB order was derived from this triplet pattern.

### 4.3. Concerted Evolution of the CRs in Nine Rhacophorids

As shown in Figure 7, the paralogous CRs of the nine studied rhacophorids were all placed in tandem (two CRs were in close proximity with only one *ND5* gene between them). Therefore, the mechanism of CR duplication may relate to the TDRL hypothesis, which is used to explain most of mitochondrial gene rearrangements [98]. This process begins with a replication error caused by slipped-strand mispairing, and one of the copied genes is then randomly excised or turned into a pseudogene. Sano et al. [20] deduced that the appearance of duplicated CRs of *Z. schlegelii* in the family Rhacophoridae resulted from the TDRL model because of the high sequence similarity and close proximity of the CRs to each other. We speculate that this model also suites our two *Polypedates* frogs as well as the other rhacophorids involved in this study (Figure 7).

Based on the principle of parsimony, the high sequence similarity resulting from concerted evolution between two CRs of rhacophorids can be attributed to a subsequent duplication-and-deletion event, as described by Kumazawa et al. [99]: a tandem replication event between two CRs produces a transient large genome with three CRs that contains two replicated gene regions between the initial two CRs, after which one of the original CRs is deleted, resulting in two remaining CRs with homogenous sequences. This extra duplicate-associated CR homogenization process also applies to the frog *Mantella madagascariensis* [18], which is phylogenetically close to the family Rhacophoridae.

Alternatively, the CR duplicates of some taxa may not be closely linked as tandem units, but rather may be far apart from each other. The TDRL mechanism mentioned above cannot be considered less plausible for such situation. However, gene recombination appears to be a more reasonable explanation. Although early studies suggested that the mitogenome is strictly matrilineal and does not undergo inter/intracellular recombination, signs of recombination have been found in several species and may contribute to some mitochondrial structural duplication [19,100,101,102]. In fact, gene recombination may be an essential part of mitochondrial DNA replication in metazoans [103]. During recombination, the sequences of the two mitogenomes are exchanged, and the portion of the exchange results in gene conversion or unequal crossover, resulting in uneven homogenized domains in the CRs [103]. For instance, this mechanism can explain the unevenness in the sequence similarity between separated CR fragments of albatrosses [104] and shorebirds [105]. Kurabayashi et al. [19] also detected duplication events in some tree frogs from the family Mantellidae with two CRs, and these events seemed to be caused by general (homologous) or illegitimate recombination. Additionally, Kurabayashi et al. [92] also suggested that homologous recombination is responsible for the concerted evolution of duplicated genes and CRs in some afrobatrachian mitogenomes. And gene conversion led by gene recombination has been used to explain the concerted evolution of paralogous CRs with high sequence similarity in a variety of organisms other than frogs, such as ticks [106], squids [66], gulper eel [28], killifishes [107], pit vipers [27,99], big-headed turtles [108], albatrosses [104], shorebirds [105] and parrots [25,109,110].

### 4.4. Causes and Advantages of Dual CRs

Unlike mitogenomes extracted from the plant [111,112] or fungal kingdoms [113,114], which contain massive introns and intergenic regions that crowd the entire genome, the mitogenomes of metazoans are commonly considered to be compact structures containing few non-coding sequences and no introns [115]. As metazoan mitogenome is subject to selective pressure favoring compactness, and duplicated genes tend to be non-functionalized and removed from the genome to achieve maximum coding efficiency and faster replication rate [27,116]. This begs the question of why such a space-occupying CR (especially in the Rhacophoridae taxa where almost all CRs are larger than the largest *ND5* gene) shows no signs of degradation when duplication occurs, and which advantages prompted additional CRs to emerge independently in a variety of lineages.

The answers to these questions may lie in the role of CRs within the mitogenome. Although the exact mechanism is still unknown, CRs have long been thought to contain the initiation site of mitogenome replication and transcription [95]. It has been proposed that multiple mitogenomes may exist in a single individual (e.g., in some molluscan species, there are male and female mitogenome types coexisting within a single cell [117]). Theoretically, an additional CR could be one more place for the initiation of transcription and replication, which could increase both the copy number and expression of mitochondrial genes, and this would make mitogenomes with duplicated CRs selectively advantageous [27]. Likewise, Shao et al. [106] also considered the example of metazoans with dual CRs and concluded that, if the replication of the mitogenome can start simultaneously at both CRs, then mitogenomes with two CRs can begin replicating at a faster rate than can mitogenomes with only one CR. Moreover, Eberhard et al. [110] found that mitogenome replication in parrots is abnormally slow, but having two CRs allows for efficient simultaneous replication and transcription from multiple loci, thus meeting the intense energy supply requirements of the bird during flight. Just as the mitochondrial genome primarily encodes proteins of the oxidative respiratory chain (also known as the electron transport chain) on the inner mitochondrial membrane, duplicated CRs have evolved in some icefishes to enhance the transcriptional and translational efficiency of the mitochondrial genes to adapt to the frigid and oxygen-rich Antarctic Ocean [118]. In addition, snakes are thermochromic animals whose body temperatures are susceptible to the effects of ambient temperature, which therefore also affect enzyme activity. Some advanced snakes with duplicated CRs have been reported to directly counteract the reduced enzymatic rate at low temperatures through transcriptional decoupling via independent CRs [119]. Almost all the species cited above have the same oxygen- and temperature-sensitive nature as anurans. As observed in these species, duplicated CRs in anurans also facilitate mitogenome replication and potentially provide greater motility for predation, reproduction, and avoidance of natural predators and adverse environmental factors. So, the presence of a duplicated CR can be maintained in the context of duplicate mitochondrial gene removal because the advantage of having a second CR can override the selection for compactness of the mitogenome.

Both tandem replication and gene conversion maintain concerted evolution between two paralogous CRs and lead to a high degree of sequence similarity [105]. Copies of CR with high sequence similarity are often considered evidence that both maintain their own functions [109]. In a study on avian mitogenome recombination, Eberhard et al. [25] suggested that duplicate CRs can only persist if the replication event produces a completely functional copy, otherwise, the incomplete copy will degrade and eventually be eliminated from the mitogenome. The concerted evolution regions (identical or nearly identical sequences) in the CRs of the nine rhacophorids involved in this study all contained intact conserved units (i.e., TAS, CSB, and O_H_ in Figure 5). Thus, all indications are that the emergence of duplicate CRs is not an intermediate transition stage in the evolutionary history of the mitogenome, but rather an ingenious evolutionary step of the rhacophorid mitogenome to stabilize and enhance its functional expression of genes.

### 4.5. Parallel Evolution of ATP8 Gene LoF in Four Polypedates and CR Duplication in Nine Rhacophorids

In our research on the phylogenetic relationships and NCR sequence alignments of four *Polypedates* species (Figure 8Ⅰ), we found a similar *ATP8* pseudogene sequence between them. Based on the solid phylogenetic relationship and sequence alignment results, we can classify the formation of the large NCR of *P. impresus* and *P. megacephalus* and the small NCR of *P. mutus* and *P. braueri* into two different evolutionary pathways. The first is that the gene *COⅡ–tRNA^Lys^* region of *P. impresus* and *P. megacephalus* first formed tandem repeats, followed by random mutations of supernumerary gene *COⅡ*, *tRNA^Lys^*, and *ATP8* (Figure 8Ⅱ). The second is that the *ATP8* genes of *P. mutus* and *P. braueri* were directly randomly mutated, forming a small NCR (Figure 8Ⅲ). The absence of *ATP8* gene in these four *Polypedates* species occurred through different pathways with different phylogenetic clusters. As parallel evolution has been found among other anuran lineages forming identical gene orders [18,120,121,122], so it indicates that the absence of *ATP8* gene in *Polypedates* frogs may have been a result of parallel evolution.

The absence of mitochondrial genes may not preclude their continued physiological functions via nuclear transport, as nuclear copies of mitochondrial sequences are found in various organisms, including invertebrates, vertebrates, fungi, and plants [123]. The lack of *ATP8* gene has been found in several metazoan species that are phylogenetically distant from frogs, such as nematodes [124], mollusks [115,125], and rotifers [126,127], most of which are invertebrates. However, among vertebrates, the absence of *ATP8* gene has only been found in the genus *Polypedates*, so this could be a distinctive characteristic that distinguishes *Polypedates* frogs from other vertebrates. The NCR left behind by the non-functionalization of the gene tends to be eliminated from the mitogenome, as the metazoan mitogenome is under strong selective pressure for genome minimization [66], and repetitive redundant sequences are likely to be rapidly deleted [27,116]. The more compact the mitogenome, the faster the self-replication rate [18].

As for the emergence of two CR copies separated by *ND5* gene in rhacophorids, it cannot be explained by a single origin because of the robust phylogenetic support for multiple origins (Figure 9). The same gene order for CR1, *ND5*, and CR2 seems to have occurred independently in two rhacophorid lineages, one of which is a common ancestor of *Z. schlegelii*, *Z. arboreus,* and *Z. omeimontis* diverging from *Z. dennysi*, while the other is a common ancestor of all four *Polypedates* frogs that directly diverged from *Zhangixalus*. This indicates duplicated CR copies in this taxon may have undergone parallel evolution too.

### 4.6. Phylogenetic Analyses and Its Insight into Gene Rearrangements of Nine Rhacophorids

In the families Heleophrynidae and Sooglossidae, we found that, consistent with previous studies based on mitochondrial and nuclear genes, Heleophrynidae were the sister taxon of all other neobatrachian frogs (ML tree topology) [11,37,128,129]. However, in most recent studies, Sooglossidae was recovered as a sister taxon to Ranoidea, which is in contrast to both of our analyses [11,37,129]. As for the families Dicroglossidae, Pyxicephalidae, and Ranidae, the monophyly of the combined Mantellidae and Rhacophoridae is not controversial, and a stable sister relationship has been shown in almost every study involving these two families [10,11,18,19,130,131,132]. However, the accurate relationships between Dicroglossinae and Ranidae have been disputed. As the sister taxon to Ranidae, as shown in our BI tree (Figure 9), Dicroglossinae can also be found in some early studies that used mitochondrial PCGs as the only phylogenetic markers [130,133]. In contrast, considering the Mantellidae + Rhacophoridae cluster as the sister group of Ranidae, as shown in our ML tree (Figure 9), was consistent with the results of recent phylogenetic studies based on multi-locus marker systems (i.e., mitochondrial and nuclear segments). The specific grouping was: (Pyxicephalidae + (Dicroglossinae + ((Mantellidae + Rhacophoridae) + Ranidae))) (also see [11] and [132]). Combining our study with others that also used mitochondrial genes highlights the advantages of using combined mitochondrial and nuclear sequences for phylogenetic purposes. This multi-marker phylogenetic reconstruction approach has also benefited taxonomic studies of other vertebrates, such as snakes [134].

Since gene arrangements are thought to reflect phylogenetic relationships [18,19,135], Zhang et al. [30] proposed that the “LTPF” cluster occurs only in the common ancestor of neobatrachians, and that it was derived from archaeobatrachians. The subsequent translocation of the *ND5* gene to the downstream of the CR occurred before the arrangement of *B. buergeri* occurrences (Figure 7), based on the basal phylogenetic position of *B. buergeri* in the family Rhacophoridae (Figure 9), and the basal phylogenetic position for genus *Buergeria* has also been determined in multiple recent phylogenetic studies [9,10,131,132]. The basal position of *B. buergeri* also corresponds to its ancestral mitochondrial gene order in the family Rhacophoridae, and the swapping of two tRNA genes (i.e., “LTPF” shuffled to “TLPF”) of the remaining eight rhacophorids can be seen as the derived conditions of *B. buergeri* (Figure 7).

We also noticed that *Z. dennysi,* which contains only a single CR, was placed at the basal position of the genus *Zhangixalus*. Considering that this was the same basal condition of *B. buergeri* and there was a single detached CR, we were able to infer that the CR duplication event that occurred in the family Rhacophoridae had multiple origins, which, as we have discussed in Section 4.5, can also be considered as a case of parallel evolution of CR duplication in *Polypedates* and *Zhangixalus* lineages. Furthermore, this CR duplication event occurred in Mantellidae, another tree frog family in Malagasy which is phylogenetically close to the family Rhacophoridae [18,19].

The sister relationships of *P. mutus*-*P. braueri* and *P. megacephalus*-*P. impresus* were strongly supported by other studies [13,14]. Combined with the fact that the formation of NCRs with different lengths in these four species also occurred in a lineage-specific manner, we can infer that *ATP8* gene LoF events occurring in the genus *Polypedates* have undergone parallel evolution in different clades (see Section 4.5).

## 5. Conclusions

In this study, the size and organization of two *Polypedates* mitogenomes were determined based on comparative mitogenomic analyses of nine rhacophorids and phylogenetic analyses of 178 anurans as well as multiple sequence alignment results. We (1) found the composition of two new mitogenomes are almost identical to other rhacophorids except with the *ATP8* gene absent; and (2) our phylogenetic analyses supporting the gene rearrangement pathway also suggested parallel evolution of the *ATP8* gene LoF in *Polypedates* and CR duplication with concerted evolution of paralogous CRs in rhacophorids. Overall, this study is a blueprint for further research on the family Rhacophoridae based on the multilocus approach, and these two new mitogenomes provide important basic data for the future research and conservation of arboreal anurans.

## Figures and Tables

**Table 1:** PCR primers used in this study.

PCR Fragments	Primer Names	Primer Nucleotide Sequences (5′-3′)	Length of Fragments
F1	COIIF ^a^	GACTCACTCAAGCGTCTATTC	~1300 bp
	ATP6R ^a^	TGTGGGCGGGTTTATT	
F2	CytbFow1 ^b^	GTYCTMCCNTGRGGHCAAATATCHTTYTG	~3500 bp
	CytbF2 ^a^	TTAGCCCTTCTATCTACCCTC	
	FND512800H ^c^	CCTATTTTDCGRATRTCYTGYTC	
	ND5R3 ^a^	CAGCCAATAAGTAAATAGGACA	
F3	ND5F2 ^a^	CTCACCCCTCTATTACGACTT	~5000 bp
	ND5Fow_sch ^b^	TGACTWGCMGCAGCAATAGAAGG	
	ND5F3 ^a^	CCCGCTGTTATGACTTGGAA	
	R16M1 ^d^	GGGTATCTAATCCCAGTTTG	
	R17N1 ^b^	GCTGAGACTTGCATGTGTAA	
	12SR1 ^a^	TTAACTTGAGTCCGCCGGTA	
	12S600H ^c^	TCGATTATAGAACAGGCTCCTCT	

^a^ Retrieved from this study; ^b^ Sano et al. [20]; ^c^ Zhang et al. [37]; ^d^ Sano et al. [36].

**Table 2:** Mitogenome nucleotide composition and skewness of nine rhacophorids.

Region	AT Content (%)										GC Content (%)								
	A	B	C	D	E	F	G	H	I		A	B	C	D	E	F	G	H	I
Genome	61.0	60.9	60.6	61.6	62.1	62.4	63.0	64.5	60.4		39.0	39.1	39.4	38.4	38.0	37.5	37.0	35.5	39.5
All PCGs	59.9	59.3	59.2	59.9	59.4	61.7	62.3	60.9	59.0		40.2	40.6	40.9	40.1	40.6	38.2	37.7	39.2	41.0
1st Codon	60.8	60.1	58.6	59.3	53.9	54.9	56.1	55.2	52.7		39.2	39.9	41.5	40.7	46.1	45.1	43.9	44.8	47.4
2nd Codon	57.5	57.2	59.6	60.5	59.6	59.5	60.5	59.6	59.6		42.5	42.8	40.5	39.5	40.4	40.5	39.4	40.5	40.4
3rd Codon	61.1	60.7	59.3	59.8	64.7	70.8	70.4	67.8	64.6		38.8	39.3	40.7	40.3	35.3	29.2	29.6	32.2	35.3
All tRNAs	57.5	57.3	57.4	57.9	58.9	59.8	59.5	58.2	56.3		42.4	42.6	42.6	42.2	41.2	40.2	40.5	41.8	43.6
All rRNAs	59.0	59.9	59.2	59.7	59.5	60.7	60.8	59.8	58.0		41.0	40.0	40.7	40.3	40.6	39.3	39.2	40.2	42.0
CR1	64.3	65.4	65.7	65.9	68.4	68.8	64.4	65.2	66.8		35.7	34.6	34.3	34.1	31.6	31.2	35.6	34.8	33.2
CR2	65.0	67.0	68.8	69.7	69.3	N/A	67.6	69.8	N/A		35.0	32.9	31.3	30.2	30.7	N/A	32.4	30.2	N/A
**Region**	**AT Skew**										**GC Skew**								
	**A**	**B**	**C**	**D**	**E**	**F**	**G**	**H**	**I**		**A**	**B**	**C**	**D**	**E**	**F**	**G**	**H**	**I**
Genome	−0.010	0.002	−0.003	−0.036	0.024	0.006	0.032	0.020	−0.010		−0.246	−0.258	−0.249	−0.214	−0.226	−0.237	−0.259	−0.251	−0.256
All PCGs	−0.085	−0.073	−0.078	−0.105	−0.044	−0.057	−0.059	−0.048	−0.078		−0.239	−0.241	−0.247	−0.197	−0.281	−0.257	−0.268	−0.286	−0.278
1st Codon	0.007	0.042	−0.085	−0.093	0.083	0.064	0.084	0.072	0.089		−0.224	−0.233	−0.210	−0.174	0.015	0.033	0.021	0.013	0.017
2nd Codon	−0.085	−0.094	−0.060	−0.107	−0.386	−0.382	−0.392	−0.386	−0.379		−0.186	−0.173	−0.284	−0.235	−0.371	−0.363	−0.371	−0.373	−0.386
3rd Codon	−0.175	−0.166	−0.086	−0.117	0.172	0.124	0.114	0.150	0.062		−0.309	−0.328	−0.248	−0.186	−0.564	−0.555	−0.561	−0.596	−0.547
All tRNAs	0.026	0.026	0.031	0.022	0.032	0.030	0.025	0.024	0.030		0.033	0.028	0.033	0.057	0.019	0.045	0.037	0.048	0.005
All rRNAs	0.146	0.145	0.146	0.128	0.181	0.174	0.191	0.188	0.146		−0.090	−0.072	−0.101	−0.076	−0.094	−0.090	−0.096	−0.106	−0.109
CR1	0.020	0.021	0.014	−0.017	−0.066	0.000	0.121	0.086	−0.010		−0.333	−0.353	−0.294	−0.308	0.032	−0.200	−0.234	−0.206	−0.184
CR2	−0.006	0.000	−0.033	−0.076	0.065	N/A	0.071	0.094	N/A		−0.326	−0.343	−0.252	−0.199	−0.173	N/A	−0.255	−0.265	N/A

A: *Polypedates impresus* (this study); B: *Polypedates mutus* (this study); C: *Polypedates megacephalus*; D: *Polypedates braueri*; E: *Zhangixalus schlegelii*; F: *Zhangixalus dennysi*; G: *Zhangixalus omeimontis*; H: *Zhangixalus arboreus*; I: *Buergeria buergeri*.

**Table 3:** Mitogenome control region (CR) sequence similarity of 16 anurans from NCBI RefSeq database with duplicated CRs.

Anurans with Duplicated CRs	GenBank ID	Length of CR1	Length of CR2	Length of Similar Regions/Similarity
*Breviceps adspersus*	NC_023379.1	6466 bp	4018 bp	3148 bp/99.0%
*Hoplobatrachus rugulosus*	NC_019615.1	1815 bp	1772 bp	1772 bp/97.2%
*Hoplobatrachus tigerinus*	NC_014581.1	3415 bp	1586 bp	1586 bp/99.2%
*Hyperolius marmoratus*	NC_023381.1	2014 bp	2286 bp	1857 bp/99.6%
*Mantella baroni*	NC_039758.1	2168 bp	2970 bp	2099 bp/94.7%
*Mantella madagascariensis*	NC_007888.1	4704 bp	2274 bp	2217 bp/93.7%
*Polypedates braueri*	NC_042797.1	1753 bp	2797 bp	1524 bp/99.9%
*Polypedates impresus*	MN869008.1	1401 bp	2264 bp	1496 bp/91.1%
*Polypedates megacephalus*	NC_043955.1	1574 bp	2230 bp	1571 bp/99.5%
*Polypedates mutus*	MN869009.1	1959 bp	2760 bp	1690 bp/99.7%
*Rana amurensis*	NC_030042.1	2695 bp	2324 bp	1708 bp/94.2%
*Rana kunyuesis*	NC_024548.1	3969 bp	2777 bp	2366 bp/96.8%
*Trichobatrachus robustus*	NC_023382.1	1699 bp	1788 bp	1390 bp/99.7%
*Zhangixalus arboreus*	LC565708.1	3617 bp	3345 bp	1718 bp/99.4%
*Zhangixalus omeimontis*	NC_046387.1	1822 bp	2214 bp	1506 bp/99.0%
*Zhangixalus schlegelii*	NC_007178.1	2688 bp	3272 bp	1510 bp/97.0%

## Data Availability

Data are contained within the article or Supplementary Materials.

## Supplementary Materials

The following supporting information can be downloaded at: https://www.mdpi.com/article/10.3390/ani12182449/s1, Figure S1. Phylogenetic tree inferred from nucleotide sequences of 13 protein-coding genes (PCGs) and two rRNA genes of the mitogenome with maximum likelihood (ML) analysis. Figure S2. Phylogenetic tree inferred from nucleotide sequences of 13 protein-coding genes (PCGs) and two rRNA genes of the mitogenome with Bayesian inference (BI) analysis. Table S1. List of species used in the phylogenetic analyses. Table S2. The best-fit nucleotide partitioning schemes and substitution models determined using PartitionFinder v2.1.1. Table S3. Mitogenome annotation of *Polypedates impresus* and *Polypedates mutus*. Table S4. Mitogenome nucleotide composition and skewness for the whole genome and individual genes of nine rhacophorids. Table S6. Mitogenome codon usage of nine rhacophorids. Table S5. Mitogenome start and stop codon usage of nine rhacophorids.

## References

[1] Boore J.L. (1999). Animal mitochondrial genomes. Nucleic Acids Res..

[2] Hjort K., Goldberg A.V., Tsaousis A.D., Hirt R.P., Embley T.M. (2010). Diversity and reductive evolution of mitochondria among microbial eukaryotes. Philos. Trans. R. Soc. B Biol. Sci..

[3] Saccone C., De Giorgi C., Gissi C., Pesole G., Reyes A. (1999). Evolutionary genomics in Metazoa: The mitochondrial DNA as a model system. Gene.

[4] Vanhove M.P., Briscoe A.G., Jorissen M.W., Littlewood D.T.J., Huyse T. (2018). The first next-generation sequencing approach to the mitochondrial phylogeny of African monogenean parasites (Platyhelminthes: Gyrodactylidae and Dactylogyridae). BMC Genom..

[5] Ramos B., González-Acuña D., Loyola D.E., Johnson W.E., Parker P.G., Massaro M., Dantas G.P., Miranda M.D., Vianna J.A. (2018). Landscape genomics: Natural selection drives the evolution of mitogenome in penguins. BMC Genom..

[6] Sharma A., Siva C., Ali S., Sahoo P.K., Nath R., Laskar M., Sarma D. (2020). The complete mitochondrial genome of the medicinal fish, *Cyprinion semiplotum*: Insight into its structural features and phylogenetic implications. Int. J. Biol. Macromol..

[7] Jiang L., Zhao L., Cheng D., Zhu L., Zhang M., Ruan Q., Chen W. (2017). The complete mitochondrial genome sequence of the Sichuan Digging Frog, *Kaloula rugifera* (Anura: Microhylidae) and its phylogenetic implications. Gene.

[8] American Museum of Natural History Amphibian Species of the World: An Online Reference, Version 6.1. https://amphibiansoftheworld.amnh.org/index.php.

[9] Chen J.-M., Prendini E., Wu Y.-H., Zhang B.-L., Suwannapoom C., Chen H.-M., Jin J.-Q., Lemmon E.M., Lemmon A.R., Stuart B.L. (2020). An integrative phylogenomic approach illuminates the evolutionary history of Old World tree frogs (Anura: Rhacophoridae). Mol. Phylogenet. Evol..

[10] Meegaskumbura M., Senevirathne G., Biju S., Garg S., Meegaskumbura S., Pethiyagoda R., Hanken J., Schneider C.J. (2015). Patterns of reproductive-mode evolution in Old World tree frogs (Anura, Rhacophoridae). Zool. Scr..

[11] Feng Y.-J., Blackburn D.C., Liang D., Hillis D.M., Wake D.B., Cannatella D.C., Zhang P. (2017). Phylogenomics reveals rapid, simultaneous diversification of three major clades of Gondwanan frogs at the Cretaceous–Paleogene boundary. Proc. Natl. Acad. Sci. USA.

[12] Brown R.M., Linkem C.W., Siler C.D., Sukumaran J., Esselstyn J.A., Diesmos A.C., Iskandar D.T., Bickford D., Evans B.J., McGuire J.A. (2010). Phylogeography and historical demography of Polypedates leucomystax in the islands of Indonesia and the Philippines: Evidence for recent human-mediated range expansion?. Mol. Phylogenet. Evol..

[13] Kuraishi N., Matsui M., Hamidy A., Belabut D.M., Ahmad N., Panha S., Sudin A., Yong H.S., Jiang J.P., Ota H. (2013). Phylogenetic and taxonomic relationships of the *Polypedates leucomystax* complex (Amphibia). Zool. Scr..

[14] Pan S., Dang N., Wang J., Zheng Y., Rao D., Li J. (2013). Molecular phylogeny supports the validity of *Polypedates impresus* Yang 2008. Asian Herpetol. Res..

[15] Amphibia China, Kunming Institute of Zoology (CAS) The Database of Chinese Amphibians. http://www.amphibiachina.org/.

[16] Alam M.S., Kurabayashi A., Hayashi Y., Sano N., Khan M.M.R., Fujii T., Sumida M. (2010). Complete mitochondrial genomes and novel gene rearrangements in two dicroglossid frogs, *Hoplobatrachus tigerinus* and *Euphlyctis hexadactylus*, from Bangladesh. Genes Genet. Syst..

[17] Yu D., Zhang J., Zheng R., Shao C. (2012). The complete mitochondrial genome of *Hoplobatrachus rugulosus* (Anura: Dicroglossidae). Mitochondrial DNA.

[18] Kurabayashi A., Usuki C., Mikami N., Fujii T., Yonekawa H., Sumida M., Hasegawa M. (2006). Complete nucleotide sequence of the mitochondrial genome of a Malagasy poison frog *Mantella madagascariensis*: Evolutionary implications on mitochondrial genomes of higher anuran groups. Mol. Phylogenet. Evol..

[19] Kurabayashi A., Sumida M., Yonekawa H., Glaw F., Vences M., Hasegawa M. (2008). Phylogeny, recombination, and mechanisms of stepwise mitochondrial genome reorganization in mantellid frogs from Madagascar. Mol. Biol. Evol..

[20] Sano N., Kurabayashi A., Fujii T., Yonekawa H., Sumida M. (2005). Complete nucleotide sequence of the mitochondrial genome of Schlegel’s tree frog *Rhacophorus schlegelii* (family Rhacophoridae): Duplicated control regions and gene rearrangements. Genes Genet. Syst..

[21] Huang A., Li H., Luo H., Ni Q., Yao Y., Xu H., Zeng B., Li Y., Wei Z., Zhang M. (2019). The complete mitochondrial genome of the tree frog, *Polypedates braueri* (Anura, Rhacophoridae). Mitochondrial DNA Part B.

[22] Huang A., Liu S., Li H., Luo H., Ni Q., Yao Y., Xu H., Zeng B., Li Y., Wei Z. (2019). The revised complete mitogenome sequence of the tree frog *Polypedates megacephalus* (Anura, Rhacophoridae) by next-generation sequencing and phylogenetic analysis. PeerJ.

[23] Fu C., Wang Q., Hu T., Lei Z., Fan H., Zhao T., Zong H. (2020). The complete mitochondrial genome of Omei Treefrog (*Rhacophorus omeimontis*). Mitochondrial DNA Part B.

[24] Inagaki H., Haramoto Y., Kubota H.Y., Shigeri Y. (2020). Complete mitochondrial genome sequence of Japanese forest green tree frog (*Rhacophorus arboreus*). Mitochondrial DNA Part B.

[25] Eberhard J.R., Wright T.F., Bermingham E. (2001). Duplication and concerted evolution of the mitochondrial control region in the parrot genus Amazona. Mol. Biol. Evol..

[26] Zheng C., Nie L., Wang J., Zhou H., Hou H., Wang H., Liu J. (2013). Recombination and evolution of duplicate control regions in the mitochondrial genome of the Asian big-headed turtle, *Platysternon megacephalum*. PLoS ONE.

[27] Kumazawa Y., Ota H., Nishida M., Ozawa T. (1996). Gene rearrangements in snake mitochondrial genomes: Highly concerted evolution of control-region-like sequences duplicated and inserted into a tRNA gene cluster. Mol. Biol. Evol..

[28] Inoue J.G., Miya M., Tsukamoto K., Nishida M. (2003). Evolution of the deep-sea gulper eel mitochondrial genomes: Large-scale gene rearrangements originated within the eels. Mol. Biol. Evol..

[29] Akiyama T., Nishida C., Momose K., Onuma M., Takami K., Masuda R. (2017). Gene duplication and concerted evolution of mitochondrial DNA in crane species. Mol. Phylogenet. Evol..

[30] Zhang P., Zhou H., Liang D., Liu Y.-F., Chen Y.-Q., Qu L.-H. (2005). The complete mitochondrial genome of a tree frog, *Polypedates megacephalus* (Amphibia: Anura: Rhacophoridae), and a novel gene organization in living amphibians. Gene.

[31] Schubert M., Lindgreen S., Orlando L. (2016). AdapterRemoval v2: Rapid adapter trimming, identification, and read merging. BMC Res. Notes.

[32] Coil D., Jospin G., Darling A.E. (2015). A5-miseq: An updated pipeline to assemble microbial genomes from Illumina MiSeq data. Bioinformatics.

[33] Bankevich A., Nurk S., Antipov D., Gurevich A.A., Dvorkin M., Kulikov A.S., Lesin V.M., Nikolenko S.I., Pham S., Prjibelski A.D. (2012). SPAdes: A new genome assembly algorithm and its applications to single-cell sequencing. J. Comput. Biol..

[34] Kurtz S., Phillippy A., Delcher A.L., Smoot M., Shumway M., Antonescu C., Salzberg S.L. (2004). Versatile and open software for comparing large genomes. Genome Biol..

[35] Walker B.J., Abeel T., Shea T., Priest M., Abouelliel A., Sakthikumar S., Cuomo C.A., Zeng Q., Wortman J., Young S.K. (2014). Pilon: An integrated tool for comprehensive microbial variant detection and genome assembly improvement. PLoS ONE.

[36] Sano N., Kurabayashi A., Fujii T., Yonekawa H., Sumida M. (2004). Complete nucleotide sequence and gene rearrangement of the mitochondrial genome of the bell-ring frog, *Buergeria buergeri* (family Rhacophoridae). Genes Genet. Syst..

[37] Zhang P., Liang D., Mao R.-L., Hillis D.M., Wake D.B., Cannatella D.C. (2013). Efficient sequencing of anuran mtDNAs and a mitogenomic exploration of the phylogeny and evolution of frogs. Mol. Biol. Evol..

[38] Bernt M., Donath A., Jühling F., Externbrink F., Florentz C., Fritzsch G., Pütz J., Middendorf M., Stadler P.F. (2013). MITOS: Improved de novo metazoan mitochondrial genome annotation. Mol. Phylogenet. Evol..

[39] Donath A., Jühling F., Al-Arab M., Bernhart S.H., Reinhardt F., Stadler P.F., Middendorf M., Bernt M. (2019). Improved annotation of protein-coding genes boundaries in metazoan mitochondrial genomes. Nucleic Acids Res..

[40] Lowe T.M., Chan P.P. (2016). tRNAscan-SE On-line: Integrating search and context for analysis of transfer RNA genes. Nucleic Acids Res..

[41] Laslett D., Canbäck B. (2008). ARWEN: A program to detect tRNA genes in metazoan mitochondrial nucleotide sequences. Bioinformatics.

[42] Huang M., Lv T., Duan R., Zhang S., Li H. (2015). The complete mitochondrial genome of *Rhacophorus dennysi* (Anura: Rhacophoridae) and phylogenetic analysis. Mitochondrial DNA Part A.

[43] Kumar S., Stecher G., Li M., Knyaz C., Tamura K. (2018). MEGA X: Molecular evolutionary genetics analysis across computing platforms. Mol. Biol. Evol..

[44] Zuker M. (2003). Mfold web server for nucleic acid folding and hybridization prediction. Nucleic Acids Res..

[45] Kerpedjiev P., Hammer S., Hofacker I.L. (2015). Forna (force-directed RNA): Simple and effective online RNA secondary structure diagrams. Bioinformatics.

[46] Benson G. (1999). Tandem repeats finder: A program to analyze DNA sequences. Nucleic Acids Res..

[47] Stothard P., Grant J.R., Van Domselaar G. (2019). Visualizing and comparing circular genomes using the CGView family of tools. Brief. Bioinform..

[48] Perna N.T., Kocher T.D. (1995). Patterns of nucleotide composition at fourfold degenerate sites of animal mitochondrial genomes. J. Mol. Evol..

[49] O’Leary N.A., Wright M.W., Brister J.R., Ciufo S., Haddad D., McVeigh R., Rajput B., Robbertse B., Smith-White B., Ako-Adjei D. (2016). Reference sequence (RefSeq) database at NCBI: Current status, taxonomic expansion, and functional annotation. Nucleic Acids Res..

[50] Zhang D., Gao F., Jakovlić I., Zou H., Zhang J., Li W.X., Wang G.T. (2020). PhyloSuite: An integrated and scalable desktop platform for streamlined molecular sequence data management and evolutionary phylogenetics studies. Mol. Ecol. Resour..

[51] Katoh K., Standley D.M. (2013). MAFFT multiple sequence alignment software version 7: Improvements in performance and usability. Mol. Biol. Evol..

[52] Ranwez V., Douzery E.J., Cambon C., Chantret N., Delsuc F. (2018). MACSE v2: Toolkit for the alignment of coding sequences accounting for frameshifts and stop codons. Mol. Biol. Evol..

[53] Talavera G., Castresana J. (2007). Improvement of phylogenies after removing divergent and ambiguously aligned blocks from protein sequence alignments. Syst. Biol..

[54] Lanfear R., Frandsen P.B., Wright A.M., Senfeld T., Calcott B. (2017). PartitionFinder 2: New methods for selecting partitioned models of evolution for molecular and morphological phylogenetic analyses. Mol. Biol. Evol..

[55] Lanfear R., Calcott B., Ho S.Y., Guindon S. (2012). PartitionFinder: Combined selection of partitioning schemes and substitution models for phylogenetic analyses. Mol. Biol. Evol..

[56] Ronquist F., Teslenko M., Van Der Mark P., Ayres D.L., Darling A., Höhna S., Larget B., Liu L., Suchard M.A., Huelsenbeck J.P. (2012). MrBayes 3.2: Efficient Bayesian phylogenetic inference and model choice across a large model space. Syst. Biol..

[57] Nguyen L.-T., Schmidt H.A., Von Haeseler A., Minh B.Q. (2015). IQ-TREE: A fast and effective stochastic algorithm for estimating maximum-likelihood phylogenies. Mol. Biol. Evol..

[58] Guindon S., Dufayard J.-F., Lefort V., Anisimova M., Hordijk W., Gascuel O. (2010). New algorithms and methods to estimate maximum-likelihood phylogenies: Assessing the performance of PhyML 3.0. Syst. Biol..

[59] Letunic I., Bork P. (2019). Interactive Tree Of Life (iTOL) v4: Recent updates and new developments. Nucleic Acids Res..

[60] Bensch S. (2000). Mitochondrial genomic rearrangements in songbirds. Mol. Biol. Evol..

[61] Ogoh K., Ohmiya Y. (2007). Concerted evolution of duplicated control regions within an ostracod mitochondrial genome. Mol. Biol. Evol..

[62] Sumida M., Kanamori Y., Kaneda H., Kato Y., Nishioka M., Hasegawa M., Yonekawa H. (2001). Complete nucleotide sequence and gene rearrangement of the mitochondrial genome of the Japanese pond frog Rana nigromaculata. Genes Genet. Syst..

[63] AmphibiaWeb University of California, Berkeley, CA, USA. https://amphibiaweb.org.

[64] Chen W.-H., Lu G., Bork P., Hu S., Lercher M.J. (2016). Energy efficiency trade-offs drive nucleotide usage in transcribed regions. Nat. Commun..

[65] Li X.-Y., Yan L.-P., Pape T., Gao Y.-Y., Zhang D. (2020). Evolutionary insights into bot flies (Insecta: Diptera: Oestridae) from comparative analysis of the mitochondrial genomes. Int. J. Biol. Macromol..

[66] Yokobori S.-I., Fukuda N., Nakamura M., Aoyama T., Oshima T. (2004). Long-term conservation of six duplicated structural genes in cephalopod mitochondrial genomes. Mol. Biol. Evol..

[67] Yamazaki N., Ueshima R., Terrett J.A., Yokobori S.-I., Kaifu M., Segawa R., Kobayashi T., Numachi K.-I., Ueda T., Nishikawa K. (1997). Evolution of pulmonate gastropod mitochondrial genomes: Comparisons of gene organizations of Euhadra, Cepaea and Albinaria and implications of unusual tRNA secondary structures. Genetics.

[68] Masta S.E., Boore J.L. (2004). The complete mitochondrial genome sequence of the spider *Habronattus oregonensis* reveals rearranged and extremely truncated tRNAs. Mol. Biol. Evol..

[69] Qiu Y., Song D., Zhou K., Sun H. (2005). The mitochondrial sequences of *Heptathela hangzhouensis* and *Ornithoctonus huwena* reveal unique gene arrangements and atypical tRNAs. J. Mol. Evol..

[70] Li R., Zhang W., Ma Z., Zhou C. (2020). Novel gene rearrangement pattern in the mitochondrial genomes of *Torleya mikhaili* and *Cincticostella fusca* (Ephemeroptera: Ephemerellidae). Int. J. Biol. Macromol..

[71] Miya M., Kawaguchi A., Nishida M. (2001). Mitogenomic exploration of higher teleostean phylogenies: A case study for moderate-scale evolutionary genomics with 38 newly determined complete mitochondrial DNA sequences. Mol. Biol. Evol..

[72] Ojala D., Montoya J., Attardi G. (1981). tRNA punctuation model of RNA processing in human mitochondria. Nature.

[73] Zhou M., Yu J., Li B., Ouyang B., Yang J. (2019). The complete mitochondrial genome of *Budorcas taxicolor tibetana* (Artiodactyla: Bovidae) and comparison with other Caprinae species: Insight into the phylogeny of the genus *Budorcas*. Int. J. Biol. Macromol..

[74] Sun C.-H., Liu H.-Y., Min X., Lu C.-H. (2020). Mitogenome of the little owl *Athene noctua* and phylogenetic analysis of Strigidae. Int. J. Biol. Macromol..

[75] Li W., Wang Z., Che Y. (2017). The complete mitogenome of the wood-feeding cockroach *Cryptocercus meridianus* (Blattodea: Cryptocercidae) and its phylogenetic relationship among cockroach Families. Int. J. Mol. Sci..

[76] Chen Z., Liu Y., Wu Y., Song F., Cai W., Li H. (2020). Novel tRNA gene rearrangements in the mitochondrial genome of *Camarochiloides weiweii* (Hemiptera: Pachynomidae). Int. J. Biol. Macromol..

[77] Xu H., Wu Y., Wang Y., Liu Z. (2020). Comparative analysis of five mitogenomes of Osmylinae (Neuroptera: Osmylidae) and their phylogenetic implications. Int. J. Biol. Macromol..

[78] Wolstenholme D.R. (1992). Animal mitochondrial DNA: Structure and evolution. Int. Rev. Cytol..

[79] Steinberg S., Cedergren R. (1994). Structural compensation in atypical mitochondrial tRNAs. Nat. Struct. Biol..

[80] Varani G., McClain W.H. (2000). The G·U wobble base pair. EMBO Rep..

[81] Lavrov D.V., Brown W.M., Boore J.L. (2000). A novel type of RNA editing occurs in the mitochondrial tRNAs of the centipede *Lithobius forficatus*. Proc. Natl. Acad. Sci. USA.

[82] Wong T.W., Clayton D.A. (1985). In vitro replication of human mitochondrial DNA: Accurate initiation at the origin of light-strand synthesis. Cell.

[83] Hixson J., Wong T., Clayton D.A. (1986). Both the conserved stem-loop and divergent 5′-flanking sequences are required for initiation at the human mitochondrial origin of light-strand DNA replication. J. Biol. Chem..

[84] Pereira S.L. (2000). Mitochondrial genome organization and vertebrate phylogenetics. Genet. Mol. Biol..

[85] Zardoya R., Garrido-Pertierra A., Bautista J.M. (1995). The complete nucleotide sequence of the mitochondrial DNA genome of the rainbow trout, *Oncorhynchus mykiss*. J. Mol. Evol..

[86] Ding L., Li W., Liao J. (2016). Mitochondrial genome of *Cricetulus migratorius* (Rodentia: Cricetidae): Insights into the characteristics of the mitochondrial genome and the phylogenetic relationships of *Cricetulus* species. Gene.

[87] Desjardins P., Morais R. (1991). Nucleotide sequence and evolution of coding and noncoding regions of a quail mitochondrial genome. J. Mol. Evol..

[88] Haring E., Kruckenhauser L., Gamauf A., Riesing M.J., Pinsker W. (2001). The complete sequence of the mitochondrial genome of *Buteo buteo* (Aves, Accipitridae) indicates an early split in the phylogeny of raptors. Mol. Biol. Evol..

[89] Nishibori M., Tsudzuki M., Hayashi T., Yamamoto Y., Yasue H. (2002). Complete nucleotide sequence of the *Coturnix chinensis* (blue-breasted quail) mitochondorial genome and a phylogenetic analysis with related species. J. Hered..

[90] Igawa T., Kurabayashi A., Usuki C., Fujii T., Sumida M. (2008). Complete mitochondrial genomes of three neobatrachian anurans: A case study of divergence time estimation using different data and calibration settings. Gene.

[91] Kurabayashi A., Yoshikawa N., Sato N., Hayashi Y., Oumi S., Fujii T., Sumida M. (2010). Complete mitochondrial DNA sequence of the endangered frog *Odorrana ishikawae* (family Ranidae) and unexpected diversity of mt gene arrangements in ranids. Mol. Phylogenet. Evol..

[92] Kurabayashi A., Sumida M. (2013). Afrobatrachian mitochondrial genomes: Genome reorganization, gene rearrangement mechanisms, and evolutionary trends of duplicated and rearranged genes. BMC Genom..

[93] Jiang D., Jiang K., Ren J., Wu J., Li J. (2019). Resurrection of the genus Leptomantis, with description of a new genus to the family Rhacophoridae (Amphibia: Anura). Asian Herpetol. Res..

[94] Madsen C.S., Ghivizzani S.C., Hauswirth W.W. (1993). Protein binding to a single termination-associated sequence in the mitochondrial DNA D-loop region. Mol. Cell. Biol..

[95] Shadel G.S., Clayton D.A. (1997). Mitochondrial DNA maintenance in vertebrates. Annu. Rev. Biochem..

[96] Kasamatsu H., Robberson D.L., Vinograd J. (1971). A novel closed-circular mitochondrial DNA with properties of a replicating intermediate. Proc. Natl. Acad. Sci. USA.

[97] San Mauro D., García-París M., Zardoya R. (2004). Phylogenetic relationships of discoglossid frogs (Amphibia: Anura: Discoglossidae) based on complete mitochondrial genomes and nuclear genes. Gene.

[98] Boore J.L. (2000). The duplication/random loss model for gene rearrangement exemplified by mitochondrial genomes of deuterostome animals. Comparative Genomics.

[99] Kumazawa Y., Ota H., Nishida M., Ozawa T. (1998). The complete nucleotide sequence of a snake (Dinodon semicarinatus) mitochondrial genome with two identical control regions. Genetics.

[100] Dowton M., Campbell N.J. (2001). Intramitochondrial recombination—Is it why some mitochondrial genes sleep around?. Trends Ecol. Evol..

[101] Endo K., Noguchi Y., Ueshima R., Jacobs H.T. (2005). Novel Repetitive Structures, Deviant Protein-Encoding Sequences andUnidentified ORFs in the Mitochondrial Genome of the Brachiopod *Lingula anatina*. J. Mol. Evol..

[102] Mueller R.L., Boore J.L. (2005). Molecular mechanisms of extensive mitochondrial gene rearrangement in plethodontid salamanders. Mol. Biol. Evol..

[103] Ladoukakis E.D., Zouros E. (2001). Recombination in animal mitochondrial DNA: Evidence from published sequences. Mol. Biol. Evol..

[104] Abbott C.L., Double M.C., Trueman J.W., Robinson A., Cockburn A. (2005). An unusual source of apparent mitochondrial heteroplasmy: Duplicate mitochondrial control regions in *Thalassarche* albatrosses. Mol. Ecol..

[105] Verkuil Y.I., Piersma T., Baker A.J. (2010). A novel mitochondrial gene order in shorebirds (Scolopacidae, Charadriiformes). Mol. Phylogenet. Evol..

[106] Shao R., Barker S.C., Mitani H., Aoki Y., Fukunaga M. (2005). Evolution of duplicate control regions in the mitochondrial genomes of metazoa: A case study with Australasian Ixodes ticks. Mol. Biol. Evol..

[107] Tatarenkov A., Avise J.C. (2007). Rapid concerted evolution in animal mitochondrial DNA. Proc. R. Soc. B Biol. Sci..

[108] Peng Q.-L., Nie L.-W., Pu Y.-G. (2006). Complete mitochondrial genome of Chinese big-headed turtle, *Platysternon megacephalum*, with a novel gene organization in vertebrate mtDNA. Gene.

[109] Schirtzinger E.E., Tavares E.S., Gonzales L.A., Eberhard J.R., Miyaki C.Y., Sanchez J.J., Hernandez A., Müeller H., Graves G.R., Fleischer R.C. (2012). Multiple independent origins of mitochondrial control region duplications in the order Psittaciformes. Mol. Phylogenet. Evol..

[110] Eberhard J.R., Wright T.F. (2016). Rearrangement and evolution of mitochondrial genomes in parrots. Mol. Phylogenet. Evol..

[111] Sloan D.B., Müller K., McCauley D.E., Taylor D.R., Štorchová H. (2012). Intraspecific variation in mitochondrial genome sequence, structure, and gene content in *Silene vulgaris*, an angiosperm with pervasive cytoplasmic male sterility. New Phytol..

[112] Skippington E., Barkman T.J., Rice D.W., Palmer J.D. (2015). Miniaturized mitogenome of the parasitic plant *Viscum scurruloideum* is extremely divergent and dynamic and has lost all nad genes. Proc. Natl. Acad. Sci. USA.

[113] Liu W., Cai Y., Zhang Q., Chen L., Shu F., Ma X., Bian Y. (2020). The mitochondrial genome of *Morchella importuna* (272.2 kb) is the largest among fungi and contains numerous introns, mitochondrial non-conserved open reading frames and repetitive sequences. Int. J. Biol. Macromol..

[114] Wang X., Song A., Wang F., Chen M., Li X., Li Q., Liu N. (2020). The 206 kbp mitochondrial genome of *Phanerochaete carnosa* reveals dynamics of introns, accumulation of repeat sequences and plasmid-derived genes. Int. J. Biol. Macromol..

[115] Gissi C., Iannelli F., Pesole G. (2008). Evolution of the mitochondrial genome of Metazoa as exemplified by comparison of congeneric species. Heredity.

[116] Rand D.M., Harrison R.G. (1986). Mitochondrial DNA transmission genetics in crickets. Genetics.

[117] Zouros E., Ball A.O., Saavedra C., Freeman K.R. (1994). An unusual type of mitochondrial DNA inheritance in the blue mussel Mytilus. Proc. Natl. Acad. Sci. USA.

[118] Zhuang X., Cheng C.-H.C. (2010). ND6 gene “lost” and found: Evolution of mitochondrial gene rearrangement in Antarctic notothenioids. Mol. Biol. Evol..

[119] Jiang Z.J., Castoe T.A., Austin C.C., Burbrink F.T., Herron M.D., McGuire J.A., Parkinson C.L., Pollock D.D. (2007). Comparative mitochondrial genomics of snakes: Extraordinary substitution rate dynamics and functionality of the duplicate control region. BMC Evol. Biol..

[120] Black W., Roehrdanz R.L. (1998). Mitochondrial gene order is not conserved in arthropods: Prostriate and metastriate tick mitochondrial genomes. Mol. Biol. Evol..

[121] Mindell D.P., Sorenson M.D., Dimcheff D.E. (1998). Multiple independent origins of mitochondrial gene order in birds. Proc. Natl. Acad. Sci. USA.

[122] Scouras A., Beckenbach K., Arndt A., Smith M.J. (2004). Complete mitochondrial genome DNA sequence for two ophiuroids and a holothuroid: The utility of protein gene sequence and gene maps in the analyses of deep deuterostome phylogeny. Mol. Phylogenet. Evol..

[123] Zhang D.-X., Hewitt G.M. (1996). Highly conserved nuclear copies of the mitochondrial control region in the desert locust Schistocerca gregaria: Some implications for population studies. Mol. Ecol..

[124] Lavrov D.V., Brown W.M. (2001). Trichinella spiralis mtDNA: A nematode mitochondrial genome that encodes a putative ATP8 and normally structured tRNAs and has a gene arrangement relatable to those of coelomate metazoans. Genetics.

[125] Uliano-Silva M., Americo J.A., Costa I., Schomaker-Bastos A., de Freitas Rebelo M., Prosdocimi F. (2016). The complete mitochondrial genome of the golden mussel *Limnoperna fortunei* and comparative mitogenomics of Mytilidae. Gene.

[126] Steinauer M.L., Nickol B.B., Broughton R., Ortí G. (2005). First sequenced mitochondrial genome from the phylum Acanthocephala (*Leptorhynchoides thecatus*) and its phylogenetic position within Metazoa. J. Mol. Evol..

[127] Suga K., Mark Welch D.B., Tanaka Y., Sakakura Y., Hagiwara A. (2008). Two circular chromosomes of unequal copy number make up the mitochondrial genome of the rotifer *Brachionus plicatilis*. Mol. Biol. Evol..

[128] Pyron R.A., Wiens J.J. (2011). A large-scale phylogeny of Amphibia including over 2800 species, and a revised classification of extant frogs, salamanders, and caecilians. Mol. Phylogenet. Evol..

[129] Irisarri I., San Mauro D., Abascal F., Ohler A., Vences M., Zardoya R. (2012). The origin of modern frogs (Neobatrachia) was accompanied by acceleration in mitochondrial and nuclear substitution rates. BMC Genom..

[130] Chen G., Wang B., Liu J., Xie F., Jiang J. (2011). Complete mitochondrial genome of *Nanorana pleskei* (Amphibia: Anura: Dicroglossidae) and evolutionary characteristics. Curr. Zool..

[131] Li J.-T., Li Y., Klaus S., Rao D.-Q., Hillis D.M., Zhang Y.-P. (2013). Diversification of rhacophorid frogs provides evidence for accelerated faunal exchange between India and Eurasia during the Oligocene. Proc. Natl. Acad. Sci. USA.

[132] Yuan Z.-Y., Zhang B.-L., Raxworthy C.J., Weisrock D.W., Hime P.M., Jin J.-Q., Lemmon E.M., Lemmon A.R., Holland S.D., Kortyna M.L. (2018). Natatanuran frogs used the Indian Plate to step-stone disperse and radiate across the Indian Ocean. Natl. Sci. Rev..

[133] Ren Z., Zhu B., Ma E., Wen J., Tu T., Cao Y., Hasegawa M., Zhong Y. (2009). Complete nucleotide sequence and gene arrangement of the mitochondrial genome of the crab-eating frog *Fejervarya cancrivora* and evolutionary implications. Gene.

[134] Li J.-N., Liang D., Wang Y.-Y., Guo P., Huang S., Zhang P. (2020). A large-scale systematic framework of Chinese snakes based on a unified multilocus marker system. Mol. Phylogenet. Evol..

[135] Macey J.R., Schulte J.A., Larson A. (2000). Evolution and phylogenetic information content of mitochondrial genomic structural features illustrated with acrodont lizards. Syst. Biol..

