# Supplementary material for: Complete Mitogenomes of Polypedates Tree Frogs Unveil Gene Rearrangement and Concerted Evolution within Rhacophoridae
Source: Animals (Basel). 2022 Sep 16;12(18):2449. doi: 10.3390/ani12182449 (PMC9494961; doi:10.3390/ani12182449)
Supplement: Supplementary file 1 [file animals-12-02449-s001.zip › Table S1. List of Species Used in the Phylogenetic Analyses.pdf]

Table S1. List of species used in the phylogenetic analyses.

No.	Class	Order	Family	Genus	Species	GenBank ID
1	Aves	Sphenisciformes	Spheniscidae	*Aptenodytes*	*Aptenodytes forsteri*	KT159230.1
2	Aves	Sphenisciformes	Spheniscidae	*Aptenodytes*	*Aptenodytes patagonicus*	MK801135.1
3	Reptilia	Crocodylia	Alligatoridae	*Alligator*	*Alligator sinensis*	AF511507.1
4	Reptilia	Crocodylia	Alligatoridae	*Alligator*	*Alligator mississippiensis*	Y13113.1
5	Amphibia	Anura	Alytidae	*Alytes*	*Alytes obstetricans pertinax*	NC_006688.1
6	Amphibia	Anura	Alytidae	*Discoglossus*	*Discoglossus galganoi*	NC_006690.1
7	Amphibia	Anura	Arthroleptidae	*Trichobatrachus*	*Trichobatrachus robustus*	NC_023382.1
8	Amphibia	Anura	Bombinatoridae	*Bombina*	*Bombina bombina*	NC_042501.1
9	Amphibia	Anura	Bombinatoridae	*Bombina*	*Bombina fortinuptialis*	NC_006402.1
10	Amphibia	Anura	Bombinatoridae	*Bombina*	*Bombina lichuanensis*	NC_021477.1
11	Amphibia	Anura	Bombinatoridae	*Bombina*	*Bombina maxima*	NC_011049.1
12	Amphibia	Anura	Bombinatoridae	*Bombina*	*Bombina microdeladigitora*	NC_021476.1
13	Amphibia	Anura	Bombinatoridae	*Bombina*	*Bombina orientalis*	NC_006689.1
14	Amphibia	Anura	Bombinatoridae	*Bombina*	*Bombina variegata*	NC_009258.1
15	Amphibia	Anura	Brevicipitidae	*Breviceps*	*Breviceps adspersus*	NC_023379.1
16	Amphibia	Anura	Bufonidae	*Anaxyrus*	*Anaxyrus americanus*	NC_047224.1
17	Amphibia	Anura	Bufonidae	*Bufo*	*Bufo gargarizans*	NC_008410.1
18	Amphibia	Anura	Bufonidae	*Bufo*	*Bufo japonicus*	NC_009886.1
19	Amphibia	Anura	Bufonidae	*Bufo*	*Bufo stejnegeri*	NC_027686.1
20	Amphibia	Anura	Bufonidae	*Bufo*	*Bufo tibetanus*	NC_020048.1
21	Amphibia	Anura	Bufonidae	*Bufotes*	*Bufotes pewzowi*	NC_047225.1
22	Amphibia	Anura	Bufonidae	*Bufotes*	*Bufotes pseudoraddei*	NC_046047.1
23	Amphibia	Anura	Bufonidae	*Bufotes*	*Bufotes sitibundus*	NC_050665.1
24	Amphibia	Anura	Bufonidae	*Duttaphrynus*	*Duttaphrynus melanostictus*	NC_005794.2
25	Amphibia	Anura	Bufonidae	*Melanophryniscus*	*Melanophryniscus moreirae*	NC_037378.1
26	Amphibia	Anura	Bufonidae	*Strauchbufo*	*Strauchbufo raddei*	NC_028424.1
27	Amphibia	Anura	Calyptocephalellidae	*Calyptocephallela*	*Calyptocephallela gayi*	NC_019997.1
28	Amphibia	Anura	Calyptocephalellidae	*Telmatobufo*	*Telmatobufo australis*	NC_027274.1
29	Amphibia	Anura	Ceratobatrachidae	*Cornufer*	*Cornufer vitianus*	NC_027671.1
30	Amphibia	Anura	Ceratophryidae	*Telmatobius*	*Telmatobius bolivianus*	NC_020002.1
31	Amphibia	Anura	Ceratophryidae	*Telmatobius*	*Telmatobius chusmisensis*	NC_030333.1
32	Amphibia	Anura	Dendrobatidae	*Anomaloglossus*	*Anomaloglossus baeobatrachus*	NC_030054.1
33	Amphibia	Anura	Dendrobatidae	*Anomaloglossus*	*Anomaloglossus blanci*	NC_037857.1
34	Amphibia	Anura	Dendrobatidae	*Anomaloglossus*	*Anomaloglossus degranvillei*	NC_037856.1
35	Amphibia	Anura	Dendrobatidae	*Anomaloglossus*	*Anomaloglossus dewynteri*	NC_037855.1
36	Amphibia	Anura	Dendrobatidae	*Anomaloglossus*	*Anomaloglossus surinamensis*	NC_037854.1
37	Amphibia	Anura	Dendrobatidae	*Hyloxalus*	*Hyloxalus subpunctatus*	NC_037379.1
38	Amphibia	Anura	Dendrobatidae	*Phyllobates*	*Phyllobates terribilis*	NC_037380.1
39	Amphibia	Anura	Dicroglossidae	*Euphlyctis*	*Euphlyctis hexadactylus*	NC_014584.1
40	Amphibia	Anura	Dicroglossidae	*Fejervarya*	*Fejervarya cancrivora*	NC_012647.1
41	Amphibia	Anura	Dicroglossidae	*Fejervarya*	*Fejervarya limnocharis*	NC_005055.1
42	Amphibia	Anura	Dicroglossidae	*Fejervarya*	*Fejervarya multistriata*	NC_029754.1
43	Amphibia	Anura	Dicroglossidae	*Hoplobatrachus*	*Hoplobatrachus rugulosus*	NC_019615.1
44	Amphibia	Anura	Dicroglossidae	*Hoplobatrachus*	*Hoplobatrachus tigerinus*	NC_014581.1
45	Amphibia	Anura	Dicroglossidae	*Limnonectes*	*Limnonectes fujianensis*	NC_007440.2
46	Amphibia	Anura	Dicroglossidae	*Nanorana*	*Nanorana parkeri*	NC_026789.1
47	Amphibia	Anura	Dicroglossidae	*Nanorana*	*Nanorana pleskei*	NC_016119.1
48	Amphibia	Anura	Dicroglossidae	*Nanorana*	*Nanorana taihangnica*	NC_024272.1
49	Amphibia	Anura	Dicroglossidae	*Nanorana*	*Nanorana ventripunctata*	NC_039094.1
50	Amphibia	Anura	Dicroglossidae	*Occidozyga*	*Occidozyga martensii*	NC_014685.1
51	Amphibia	Anura	Dicroglossidae	*Quasipaa*	*Quasipaa boulengeri*	NC_021937.1
52	Amphibia	Anura	Dicroglossidae	*Quasipaa*	*Quasipaa spinosa*	NC_013270.1
53	Amphibia	Anura	Dicroglossidae	*Quasipaa*	*Quasipaa yei*	NC_024843.1
54	Amphibia	Anura	Heleophrynidae	*Heleophryne*	*Heleophryne regis*	NC_019998.1
55	Amphibia	Anura	Hemisotidae	*Hemisus*	*Hemisus marmoratus*	NC_023380.1
56	Amphibia	Anura	Hylidae	*Bokermannohyla*	*Bokermannohyla alvarengai*	NC_036493.1
57	Amphibia	Anura	Hylidae	*Dryophytes*	*Dryophytes japonicus*	NC_010232.1
58	Amphibia	Anura	Hylidae	*Dryophytes*	*Dryophytes suweonensis*	NC_032380.1
59	Amphibia	Anura	Hylidae	*Dryophytes*	*Dryophytes versicolor*	NC_045917.1
60	Amphibia	Anura	Hylidae	*Hyla*	*Hyla annectans*	NC_025309.1

Table S1. *Cont.*

No.	Class	Order	Family	Genus	Species	GenBank ID
61	Amphibia	Anura	Hylidae	*Hyla*	*Hyla chinensis*	NC_006403.1
62	Amphibia	Anura	Hylidae	*Hyla*	*Hyla tsinlingensis*	NC_026524.1
63	Amphibia	Anura	Hylidae	*Hyla*	*Hyla ussuriensis*	NC_029410.1
64	Amphibia	Anura	Hylidae	*Pseudis*	*Pseudis tocantins*	NC_041426.1
65	Amphibia	Anura	Hyperoliidae	*Hyperolius*	*Hyperolius marmoratus*	NC_023381.1
66	Amphibia	Anura	Leiopelmatidae	*Leiopelma*	*Leiopelma archeyi*	NC_014691.1
67	Amphibia	Anura	Leiopelmatidae	*Leiopelma*	*Leiopelma hochstetteri*	NC_027072.1
68	Amphibia	Anura	Limnodynastidae	*Lechriodus*	*Lechriodus melanopyga*	NC_019999.1
69	Amphibia	Anura	Mantellidae	*Mantella*	*Mantella baroni*	NC_039758.1
70	Amphibia	Anura	Mantellidae	*Mantella*	*Mantella madagascariensis*	NC_007888.1
71	Amphibia	Anura	Megophryidae	*Leptobrachella*	*Leptobrachella oshanensis*	NC_020610.1
72	Amphibia	Anura	Megophryidae	*Leptobrachium*	*Leptobrachium boringii*	NC_024427.1
73	Amphibia	Anura	Megophryidae	*Leptobrachium*	*Leptobrachium leishanense*	NC_031411.1
74	Amphibia	Anura	Megophryidae	*Megophrys*	*Megophrys shapingensis*	NC_018785.1
75	Amphibia	Anura	Megophryidae	*Oreolalax*	*Oreolalax lichuanensis*	NC_030627.1
76	Amphibia	Anura	Megophryidae	*Oreolalax*	*Oreolalax major*	NC_030605.1
77	Amphibia	Anura	Megophryidae	*Oreolalax*	*Oreolalax multipunctatus*	NC_037382.1
78	Amphibia	Anura	Megophryidae	*Oreolalax*	*Oreolalax omeimontis*	NC_049862.1
79	Amphibia	Anura	Megophryidae	*Scutiger*	*Scutiger ningshanensis*	NC_031426.1
80	Amphibia	Anura	Microhylidae	*Kaloula*	*Kaloula borealis*	NC_020044.1
81	Amphibia	Anura	Microhylidae	*Kaloula*	*Kaloula pulchra*	NC_006405.1
82	Amphibia	Anura	Microhylidae	*Kaloula*	*Kaloula rugifera*	NC_029409.1
83	Amphibia	Anura	Microhylidae	*Kaloula*	*Kaloula verrucosa*	NC_039411.1
84	Amphibia	Anura	Microhylidae	*Microhyla*	*Microhyla butleri*	NC_030049.1
85	Amphibia	Anura	Microhylidae	*Microhyla*	*Microhyla fissipes*	NC_045110.1
86	Amphibia	Anura	Microhylidae	*Microhyla*	*Microhyla heymonsi*	NC_006406.1
87	Amphibia	Anura	Microhylidae	*Microhyla*	*Microhyla mixtura*	NC_038130.1
88	Amphibia	Anura	Microhylidae	*Microhyla*	*Microhyla okinavensis*	NC_010233.1
89	Amphibia	Anura	Microhylidae	*Microhyla*	*Microhyla ornata*	NC_009422.1
90	Amphibia	Anura	Microhylidae	*Microhyla*	*Microhyla pulchra*	NC_024547.1
91	Amphibia	Anura	Microhylidae	*Microhyla*	*Microhyla taraiensis*	NC_039176.1
92	Amphibia	Anura	Pelobatidae	*Pelobates*	*Pelobates cultripes*	NC_008144.1
93	Amphibia	Anura	Pelobatidae	*Pelobates*	*Pelobates fuscus*	NC_051953.1
94	Amphibia	Anura	Pelodytidae	*Pelodytes*	*Pelodytes cf. punctatus II-2011*	NC_020000.1
95	Amphibia	Anura	Pipidae	*Hymenochirus*	*Hymenochirus boettgeri*	NC_015615.1
96	Amphibia	Anura	Pipidae	*Pipa*	*Pipa carvalhoi*	NC_015617.1
97	Amphibia	Anura	Pipidae	*Pseudhymenochirus*	*Pseudhymenochirus merlini*	NC_015618.1
98	Amphibia	Anura	Pipidae	*Xenopus*	*Xenopus allofraseri*	NC_044874.1
99	Amphibia	Anura	Pipidae	*Xenopus*	*Xenopus amieti*	NC_044876.1
100	Amphibia	Anura	Pipidae	*Xenopus*	*Xenopus andrei*	NC_044878.1
101	Amphibia	Anura	Pipidae	*Xenopus*	*Xenopus borealis*	NC_018776.1
102	Amphibia	Anura	Pipidae	*Xenopus*	*Xenopus boumbaensis*	NC_044877.1
103	Amphibia	Anura	Pipidae	*Xenopus*	*Xenopus calcaratus*	NC_044865.1
104	Amphibia	Anura	Pipidae	*Xenopus*	*Xenopus clivii*	NC_044886.1
105	Amphibia	Anura	Pipidae	*Xenopus*	*Xenopus epitropicalis*	NC_044867.1
106	Amphibia	Anura	Pipidae	*Xenopus*	*Xenopus eysoole*	NC_044884.1
107	Amphibia	Anura	Pipidae	*Xenopus*	*Xenopus fischbergi*	NC_044888.1
108	Amphibia	Anura	Pipidae	*Xenopus*	*Xenopus gilli*	NC_044871.1
109	Amphibia	Anura	Pipidae	*Xenopus*	*Xenopus itombwensis*	NC_044879.1
110	Amphibia	Anura	Pipidae	*Xenopus*	*Xenopus kobeli*	NC_044883.1
111	Amphibia	Anura	Pipidae	*Xenopus*	*Xenopus laevis*	NC_001573.1
112	Amphibia	Anura	Pipidae	*Xenopus*	*Xenopus largeni*	NC_044868.1
113	Amphibia	Anura	Pipidae	*Xenopus*	*Xenopus lenduensis*	NC_044880.1
114	Amphibia	Anura	Pipidae	*Xenopus*	*Xenopus longipes*	NC_044885.1
115	Amphibia	Anura	Pipidae	*Xenopus*	*Xenopus mellotropicalis*	NC_044866.1
116	Amphibia	Anura	Pipidae	*Xenopus*	*Xenopus muelleri*	NC_044887.1
117	Amphibia	Anura	Pipidae	*Xenopus*	*Xenopus parafraseri*	NC_044872.1
118	Amphibia	Anura	Pipidae	*Xenopus*	*Xenopus petersii*	NC_044869.1
119	Amphibia	Anura	Pipidae	*Xenopus*	*Xenopus poweri*	NC_044870.1
120	Amphibia	Anura	Pipidae	*Xenopus*	*Xenopus pygmaeus*	NC_044873.1

Table S1. *Cont.*

No.	Class	Order	Family	Genus	Species	GenBank ID
121	Amphibia	Anura	Pipidae	*Xenopus*	*Xenopus ruwenzoriensis*	NC_044882.1
122	Amphibia	Anura	Pipidae	*Xenopus*	*Xenopus tropicalis*	NC_006839.1
123	Amphibia	Anura	Pipidae	*Xenopus*	*Xenopus vestitus*	NC_044881.1
124	Amphibia	Anura	Pipidae	*Xenopus*	*Xenopus victorianus*	NC_018775.1
125	Amphibia	Anura	Pipidae	*Xenopus*	*Xenopus wittei*	NC_044875.1
126	Amphibia	Anura	Pyxicephalidae	*Pyxicephalus*	*Pyxicephalus adspersus*	NC_044480.1
127	Amphibia	Anura	Ranidae	*Amolops*	*Amolops granulosus*	NC_044901.1
128	Amphibia	Anura	Ranidae	*Amolops*	*Amolops loloensis*	NC_029250.1
129	Amphibia	Anura	Ranidae	*Amolops*	*Amolops mantzorum*	NC_024180.1
130	Amphibia	Anura	Ranidae	*Amolops*	*Amolops ricketti*	NC_023949.1
131	Amphibia	Anura	Ranidae	*Amolops*	*Amolops wuyiensis*	NC_025591.1
132	Amphibia	Anura	Ranidae	*Babina*	*Babina adenopleura*	NC_018771.1
133	Amphibia	Anura	Ranidae	*Babina*	*Babina holsti*	NC_022870.1
134	Amphibia	Anura	Ranidae	*Babina*	*Babina subaspera*	NC_022871.1
135	Amphibia	Anura	Ranidae	*Glandirana*	*Glandirana emeljanovi*	NC_030211.1
136	Amphibia	Anura	Ranidae	*Glandirana*	*Glandirana tientaiensis*	NC_025226.1
137	Amphibia	Anura	Ranidae	*Lithobates*	*Lithobates catesbeianus*	NC_022696.1
138	Amphibia	Anura	Ranidae	*Lithobates*	*Lithobates okaloosae*	NC_028283.1
139	Amphibia	Anura	Ranidae	*Lithobates*	*Lithobates sylvaticus*	NC_027236.1
140	Amphibia	Anura	Ranidae	*Nidirana*	*Nidirana okinavana*	NC_022872.1
141	Amphibia	Anura	Ranidae	*Odorrana*	*Odorrana graminea*	NC_050884.1
142	Amphibia	Anura	Ranidae	*Odorrana*	*Odorrana hainanensis*	NC_034984.1
143	Amphibia	Anura	Ranidae	*Odorrana*	*Odorrana ishikawae*	NC_015305.1
144	Amphibia	Anura	Ranidae	*Odorrana*	*Odorrana livida*	NC_043768.1
145	Amphibia	Anura	Ranidae	*Odorrana*	*Odorrana margaretae*	NC_024603.1
146	Amphibia	Anura	Ranidae	*Odorrana*	*Odorrana schmackeri*	NC_027827.1
147	Amphibia	Anura	Ranidae	*Odorrana*	*Odorrana tormota*	NC_009423.1
148	Amphibia	Anura	Ranidae	*Odorrana*	*Odorrana wuchuanensis*	NC_034983.1
149	Amphibia	Anura	Ranidae	*Pelophylax*	*Pelophylax bedriagae*	NC_029200.1
150	Amphibia	Anura	Ranidae	*Pelophylax*	*Pelophylax cf. bedriagae AFL1*	NC_029196.1
151	Amphibia	Anura	Ranidae	*Pelophylax*	*Pelophylax cf. bedriagae GM178*	NC_029201.1
152	Amphibia	Anura	Ranidae	*Pelophylax*	*Pelophylax cf. bedriagae MPFC1082*	NC_029197.1
153	Amphibia	Anura	Ranidae	*Pelophylax*	*Pelophylax cf. terentievi GM87-239*	NC_029198.1
154	Amphibia	Anura	Ranidae	*Pelophylax*	*Pelophylax cf. terentievi MPFC1736*	NC_029199.1
155	Amphibia	Anura	Ranidae	*Pelophylax*	*Pelophylax chosenicus*	NC_016059.1
156	Amphibia	Anura	Ranidae	*Pelophylax*	*Pelophylax cretensis*	NC_025575.1
157	Amphibia	Anura	Ranidae	*Pelophylax*	*Pelophylax cypriensis*	NC_026893.1
158	Amphibia	Anura	Ranidae	*Pelophylax*	*Pelophylax epeiroticus*	NC_026894.1
159	Amphibia	Anura	Ranidae	*Pelophylax*	*Pelophylax kurtmuelleri*	NC_026895.1
160	Amphibia	Anura	Ranidae	*Pelophylax*	*Pelophylax nigromaculatus*	NC_002805.1
161	Amphibia	Anura	Ranidae	*Pelophylax*	*Pelophylax plancyi*	NC_009264.1
162	Amphibia	Anura	Ranidae	*Pelophylax*	*Pelophylax shqipericus*	NC_026896.1
163	Amphibia	Anura	Ranidae	*Rana*	*Rana amurensis*	NC_030042.1
164	Amphibia	Anura	Ranidae	*Rana*	*Rana cf. chensinensis QYCRCCH_C*	NC_023529.1
165	Amphibia	Anura	Ranidae	*Rana*	*Rana chaochiaoensis*	NC_035803.1
166	Amphibia	Anura	Ranidae	*Rana*	*Rana draytonii*	NC_028296.1
167	Amphibia	Anura	Ranidae	*Rana*	*Rana dybowskii*	NC_023528.1
168	Amphibia	Anura	Ranidae	*Rana*	*Rana huanrensis*	NC_028521.1
169	Amphibia	Anura	Ranidae	*Rana*	*Rana kukunoris*	NC_035804.1
170	Amphibia	Anura	Ranidae	*Rana*	*Rana kunyuensis*	NC_024548.1
171	Amphibia	Anura	Ranidae	*Rana*	*Rana omeimontis*	NC_035805.1
172	Amphibia	Anura	Ranidae	*Rana*	*Rana temporaria*	NC_042226.1
173	Amphibia	Anura	Ranidae	*Sylvirana*	*Sylvirana guentheri*	NC_024748.1
174	Amphibia	Anura	Rhacophoridae	*Buergeria*	*Buergeria buergeri*	NC_008975.1
175	Amphibia	Anura	Rhacophoridae	*Polypedates*	*Polypedates braueri*	NC_042797.1
176	Amphibia	Anura	Rhacophoridae	*Polypedates*	*Polypedates megacephalus*	NC_043955.1
**177**	**Amphibia**	**Anura**	**Rhacophoridae**	***Polypedates***	***Polypedates impresus* (this study)**	**MN869008.1**
**178**	**Amphibia**	**Anura**	**Rhacophoridae**	***Polypedates***	***Polypedates mutus* (this study)**	**MN869009.1**
179	Amphibia	Anura	Rhacophoridae	*Zhangixalus*	*Zhangixalus arboreus*	LC565708.1
180	Amphibia	Anura	Rhacophoridae	*Zhangixalus*	*Zhangixalus dennysi*	NC_027452.1

Table S1. *Cont.*

No.	Class	Order	Family	Genus	Species	GenBank ID
181	Amphibia	Anura	Rhacophoridae	*Zhangixalus*	*Zhangixalus omeimontis*	NC_046387.1
182	Amphibia	Anura	Rhacophoridae	*Zhangixalus*	*Zhangixalus schlegelii*	NC_007178.1
183	Amphibia	Anura	Rhinophrynidae	*Rhinophrynus*	*Rhinophrynus dorsalis*	NC_015620.1
184	Amphibia	Anura	Scaphiopodidae	*Scaphiopus*	*Scaphiopus holbrookii*	NC_037377.1
185	Amphibia	Anura	Sooglossidae	*Sooglossus*	*Sooglossus thomasseti*	NC_020001.1
