# Supplementary material for: Complete Mitogenomes of Polypedates Tree Frogs Unveil Gene Rearrangement and Concerted Evolution within Rhacophoridae
Source: Animals (Basel). 2022 Sep 16;12(18):2449. doi: 10.3390/ani12182449 (PMC9494961; doi:10.3390/ani12182449)
Supplement: Supplementary file 1 [file animals-12-02449-s001.zip › Table S2. Partitioning Schemes and Substitution Models.pdf]

**Table S2.** The best-fit nucleotide partitioning schemes and substitution models determined using PartitionFinder v2.1.1.

| Subsets | Subset Partitions | Best Model |
|---|---|---|
| Subset 1 | *16S rRNA* | GTR+I+G |
| Subset 2 | *12S rRNA* | GTR+I+G |
| Subset 3 | *ATP6* 1st Codon | GTR+I+G |
| Subset 4 | *ATP6* 2nd Codon | GTR+I+G |
| Subset 5 | *ATP6* 3rd Codon | GTR+G |
| Subset 6 | *ATP8* 1st Codon, *ATP8* 2nd Codon | GTR+I+G |
| Subset 7 | *ATP8* 3rd Codon, *COIII* 3rd Codon | GTR+I+G |
| Subset 8 | *COI* 1st Codon | SYM+I+G |
| Subset 9 | *COIII* 2nd Codon, *COI* 2nd Codon | GTR+I+G |
| Subset 10 | *COI* 3rd Codon | GTR+G |
| Subset 11 | *COII* 1st Codon | GTR+I+G |
| Subset 12 | *COII* 2nd Codon | GTR+I+G |
| Subset 13 | *COII* 3rd Codon | GTR+G |
| Subset 14 | *COIII* 1st Codon | SYM+I+G |
| Subset 15 | *CYTB* 1st Codon | GTR+I+G |
| Subset 16 | *ND1* 2nd Codon, *CYTB* 2nd Codon | GTR+I+G |
| Subset 17 | *CYTB* 3rd Codon | GTR+I+G |
| Subset 18 | *ND1* 1st Codon | GTR+I+G |
| Subset 19 | *ND1* 3rd Codon | GTR+G |
| Subset 20 | *ND2* 1st Codon | GTR+I+G |
| Subset 21 | *ND2* 2nd Codon | GTR+I+G |
| Subset 22 | *ND2* 3rd Codon | GTR+G |
| Subset 23 | *ND3* 1st Codon | SYM+I+G |
| Subset 24 | *ND3* 2nd Codon | GTR+I+G |
| Subset 25 | *ND3* 3rd Codon | GTR+I+G |
| Subset 26 | *ND4L* 1st Codon | GTR+G |
| Subset 27 | *ND4L* 2nd Codon | GTR+G |
| Subset 28 | *ND4* 3rd Codon, *ND4L* 3rd Codon | GTR+I+G |
| Subset 29 | *ND4* 1st Codon | GTR+I+G |
| Subset 30 | *ND4* 2nd Codon, *ND5* 2nd Codon | GTR+I+G |
| Subset 31 | *ND5* 1st Codon | GTR+I+G |
| Subset 32 | *ND5* 3rd Codon | GTR+G |
| Subset 33 | *ND6* 1st Codon | GTR+I+G |
| Subset 34 | *ND6* 2nd Codon | GTR+I+G |
| Subset 35 | *ND6* 3rd Codon | GTR+G |
