# Supplementary material for: Complete Mitogenomes of Polypedates Tree Frogs Unveil Gene Rearrangement and Concerted Evolution within Rhacophoridae
Source: Animals (Basel). 2022 Sep 16;12(18):2449. doi: 10.3390/ani12182449 (PMC9494961; doi:10.3390/ani12182449)
Supplement: Supplementary file 1 [file animals-12-02449-s001.zip › Table S3. Mitogenome Annotation.pdf]

Table S3. Mitogenome annotation of *Polypedates impresus* and *Polypedates mutus*.

Features	Strand	Position		Length		Intergenic Nucleotides		Start codon - Stop codon		Anticodon
		P. Impresus	***P.mutus***	***P. Impresus***	***P.mutus***	***P. Impresus***	***P.mutus***	***P. Impresus***	***P.mutus***	
$tRNA^{Thr}$	H	1-71	1-71	71	71	-	-	-	-	TGT
$tRNA^{Leu(CUN)}$	H	72-142	72-143	71	72	5	5	-	-	TAG
$tRNA^{Pro}$	L	148-216	149-217	69	69	1	1	-	-	TGG
$tRNA^{Phe}$	H	218-287	219-288	70	70	-2	-2	-	-	GAA
12S rRNA	H	286-1215	287-1217	930	931	-	-	-	-	-
$tRNA^{Val}$	H	1216-1284	1218-1286	69	69	-	-	-	-	TAC
16S rRNA	H	1285-2857	1287-2859	1573	1573	2	2	-	-	-
$tRNA^{Leu(UUR)}$	H	2856-2929	2862-2935	74	74	3	3	-	-	TAA
ND1	H	2933-3893	2939-3899	961	961	-	-	ATG -T(AA)	ATG - T(AA)	-
$tRNA^{Ile}$	H	3894-3964	3900-3970	71	71	-1	-1	-	-	GAT
$tRNA^{Gln}$	L	3964-4034	3970-4040	71	71	-1	-1	-	-	TTG
$tRNA^{Met}$	H	4034-4102	4040-4108	69	69	-	-	-	-	CAT
ND2	H	4103-5140	4109-5146	1038	1038	-2	-2	ATT - TAG	ATT - TAG	-
$tRNA^{Trp}$	H	5139-5209	5145-5215	71	71	-	-	-	-	TCA
$tRNA^{Ala}$	L	5210-5279	5216-5285	70	70	1	1	-	-	TGC
$tRNA^{Asn}$	L	5281-5353	5287-5359	73	73	-	-	-	-	GTT
O_L	H	5354-5384	5360-5390	31	30	-3	-3	-	-	-
$tRNA^{Cys}$	L	5381-5445	5388-5452	65	65	-	-	-	-	GCA
$tRNA^{Tyr}$	L	5446-5512	5453-5519	67	67	4	4	-	-	GTA
COI	H	5517-7070	5524-7077	1554	1554	-13	-13	ATA - AGG	ATA - AGG	-
$tRNA^{Ser(UCN)}$	L	7058-7128	7065-7135	71	71	1	1	-	-	TGA
$tRNA^{Asp}$	H	7130-7198	7137-7205	69	69	-	-	-	-	GTC
COII	H	7199-7888	7206-7895	690	690	5	5	ATG - TAA	ATG - TAA	-
$tRNA^{Lys}$	H	7894-7963	7901-7971	71	71	-	-	-	-	TTT
NCR	H	7964-8832	7972-8120	869	149	-	-	-	-	-
ATP6	H	8833-9511	8121-8799	679	679	-	-	ATA - T(AA)	ATA - T(AA)	-
COIII	H	9512-10295	8800-9583	784	784	-	-	ATG - T(AA)	ATG - T(AA)	-
$tRNA^{Gly}$	H	10296-10363	9584-9651	68	68	-	-	-	-	TCC

Table S3. *Cont.*

Features	Strand	Position		Length		Intergenic Nucleotides		Start codon - Stop codon		Anticodon
		P. Impresus	***P.mutus***	***P. Impresus***	***P.mutus***	***P. Impresus***	***P.mutus***	***P. Impresus***	***P.mutus***	
ND3	H	10364-10703	9652-9991	340	340	-	-	ATG - T(AA)	ATG - T(AA)	-
tRNA^{Arg}	H	10704-10772	9992-10060	69	69	-	1	-	-	TCG
ND4L	H	10773-11057	10062-10346	285	285	-7	-7	ATG - TAA	ATG - TAA	-
ND4	H	11051-12413	10340-11702	1363	1363	-	-	GTG - T(AA)	ATG - T(AA)	-
tRNA^{His}	H	12414-12482	11703-11771	69	69	-	-	-	-	GTG
tRNA$^{Ser(AGY)}$	H	12483-12550	11772-11839	68	68	2	2	-	-	GCT
ND6	L	12553-13044	11842-12333	492	492	-	-	ATG - AGG	ATG - AGG	-
tRNA^{Glu}	L	13045-13113	12334-12401	68	68	4	4	-	-	TTC
CYTB	H	13118-14273	12406-13558	1156	1153	-	-	ATG - T(AA)	ATG - T(AA)	-
CR1	H	14274-15674	13559-15517	1401	1959	-	-	-	-	-
ND5	H	15675-17456	15518-17296	1782	1779	-	-	ATG - TAA	ATG - TAA	-
CR2	H	17457-19720	17297-20056	2264	2760	-	-	-	-	-

The letters "H" and "L" stand for heavy and light strands, respectively. A positive value denotes the base-pair spacing between genes, whereas a negative number indicates that the base-pair intervals between genes are overlapping.
