# Supplementary material for: Complete Mitogenomes of Polypedates Tree Frogs Unveil Gene Rearrangement and Concerted Evolution within Rhacophoridae
Source: Animals (Basel). 2022 Sep 16;12(18):2449. doi: 10.3390/ani12182449 (PMC9494961; doi:10.3390/ani12182449)
Supplement: Supplementary file 1 [file animals-12-02449-s001.zip › Table S4. Mitogenome Nucleotide Composition and Skewness.pdf]

Table S4. Mitogenome nucleotide composition and skewness for the whole genome and individual genes of nine rhacophorids.

Region	Species	A%	T(U)%	G%	C%	A+T%	G+C%	AT skew	GC skew
Whole-Genome	A	30.2	30.8	14.7	24.3	61.0	39.0	-0.010	-0.246
	B	30.5	30.4	14.5	24.6	60.9	39.1	0.002	-0.258
	C	30.2	30.4	14.8	24.6	60.6	39.4	-0.003	-0.249
	D	29.7	31.9	15.1	23.3	61.6	38.4	-0.036	-0.214
	E	31.8	30.3	14.7	23.3	62.1	38.0	0.024	-0.226
	F	31.4	31.0	14.3	23.2	62.4	37.5	0.006	-0.237
	G	32.5	30.5	13.7	23.3	63.0	37.0	0.032	-0.259
	H	32.9	31.6	13.3	22.2	64.5	35.5	0.020	-0.251
	I	29.9	30.5	14.7	24.8	60.4	39.5	-0.010	-0.256
12S rRNA	A	33.0	24.2	19.6	23.2	57.2	42.8	0.154	-0.084
	B	33.1	23.5	20.2	23.2	56.6	43.4	0.170	-0.069
	C	32.9	24.8	19.4	22.9	57.7	42.3	0.140	-0.083
	D	33.0	24.0	20.5	22.6	57.0	43.1	0.158	-0.049
	E	32.6	24.4	19.9	23.2	57.0	43.1	0.144	-0.075
	F	33.8	24.1	19.7	22.3	57.9	42.0	0.167	-0.062
	G	33.6	23.2	19.3	23.9	56.8	43.2	0.183	-0.105
	H	32.8	23.7	19.7	23.9	56.5	43.6	0.161	-0.097
	I	32.7	23.0	20.0	24.4	55.7	44.4	0.174	-0.112
16S rRNA	A	34.4	25.7	18.1	21.9	60.1	40.0	0.145	-0.095
	B	35.1	26.9	17.6	20.4	62.0	38.0	0.132	-0.074
	C	34.6	25.6	17.7	22.2	60.2	39.9	0.150	-0.113
	D	34.1	27.3	17.5	21.2	61.4	38.7	0.111	-0.096
	E	36.7	24.4	17.4	21.6	61.1	39.0	0.202	-0.106
	F	36.7	25.6	16.8	20.9	62.3	37.7	0.178	-0.109
	G	37.7	25.4	16.8	20.1	63.1	36.9	0.196	-0.089
	H	37.1	24.6	17.0	21.3	61.7	38.3	0.202	-0.113
	I	33.5	25.8	18.0	22.7	59.3	40.7	0.131	-0.116
ND1	A	29.3	32.6	12.9	25.2	61.9	38.1	-0.053	-0.323
	B	29.3	32.5	12.8	25.4	61.8	38.2	-0.052	-0.330
	C	28.7	32.3	13.0	26.0	61.0	39.0	-0.059	-0.333
	D	28.0	33.0	14.0	25.0	61.0	39.0	-0.082	-0.282
	E	29.0	32.0	13.3	25.6	61.0	38.9	-0.049	-0.316
	F	29.4	32.5	13.1	25.0	61.9	38.1	-0.049	-0.311
	G	30.3	33.9	12.3	23.5	64.2	35.8	-0.057	-0.314
	H	30.1	32.4	12.3	25.3	62.5	37.6	-0.037	-0.346
	I	26.2	34.1	13.1	26.6	60.3	39.7	-0.131	-0.342

Table S4. *Cont.*

Region	Species	A%	T(U)%	G%	C%	A+T%	G+C%	AT skew	GC skew
	A	32.0	32.2	10.9	25.0	64.2	35.9	-0.003	-0.393
	B	31.3	31.8	10.9	26.0	63.1	36.9	-0.008	-0.409
	C	31.7	31.4	11.2	25.7	63.1	36.9	0.005	-0.394
	D	30.5	32.5	11.8	25.1	63.0	36.9	-0.032	-0.360
ND2	E	32.2	28.4	12.2	27.2	60.6	39.4	0.062	-0.379
	F	34.8	30.0	9.9	25.3	64.8	35.2	0.074	-0.437
	G	34.4	30.7	10.5	24.4	65.1	34.9	0.056	-0.398
	H	33.3	30.2	11.1	25.3	63.5	36.4	0.049	-0.389
	I	32.4	30.1	9.8	27.7	62.5	37.5	0.037	-0.477
	A	23.6	33.1	18.4	24.8	56.7	43.2	-0.168	-0.148
	B	24.6	32.1	18.5	24.8	56.7	43.3	-0.132	-0.145
	C	23.4	32.8	18.6	25.2	56.2	43.8	-0.168	-0.150
	D	24.0	33.3	19.0	23.6	57.3	42.6	-0.162	-0.108
COI	E	26.7	33.1	17.0	23.2	59.8	40.2	-0.107	-0.155
	F	26.6	33.4	16.9	23.2	60.0	40.1	-0.114	-0.158
	G	26.5	34.9	16.8	21.8	61.4	38.6	-0.137	-0.130
	H	26.7	33.6	16.6	23.1	60.3	39.7	-0.114	-0.162
	I	24.8	32.6	17.9	24.8	57.4	42.7	-0.135	-0.162
	A	30.9	29.9	15.8	23.5	60.8	39.3	0.016	-0.196
	B	32.3	30.6	15.1	22.0	62.9	37.1	0.027	-0.186
	C	30.1	29.3	16.5	24.1	59.4	40.6	0.013	-0.187
	D	31.0	28.8	17.1	23.0	59.8	40.1	0.037	-0.147
COII	E	32.3	27.9	15.6	24.1	60.2	39.7	0.073	-0.213
	F	33.0	30.4	15.2	21.3	63.4	36.5	0.041	-0.168
	G	32.3	31.0	15.2	21.5	63.3	36.7	0.021	-0.171
	H	31.9	29.4	15.8	23.0	61.3	38.8	0.041	-0.185
	I	31.3	27.9	15.0	25.8	59.2	40.8	0.057	-0.264
	A	*ATP8* gene was absent in *Polypedates impresus.*							
	B	*ATP8* gene was absent in *Polypedates mutus.*							
	C	*ATP8* gene was absent in *Polypedates megacephalus.*							
	D	*ATP8* gene was absent in *Polypedates braueri.*							
ATP8	E	33.3	29.7	9.7	27.3	63.0	37.0	0.058	-0.475
	F	35.8	33.9	6.7	23.6	69.7	30.3	0.026	-0.56
	G	35.2	36.4	6.8	21.6	71.6	28.4	-0.017	-0.522
	H	37.0	32.7	7.3	23.0	69.7	30.3	0.061	-0.52
	I	35.8	30.3	8.5	25.5	66.1	34.0	0.083	-0.512

Table S4. *Cont.*

Region	Species	A%	T(U)%	G%	C%	A+T%	G+C%	AT skew	GC skew
ATP6	A	27.2	35.5	11.8	25.5	62.7	37.3	-0.132	-0.367
	B	27.5	33.4	11.8	27.2	60.9	39.0	-0.097	-0.395
	C	27.1	33.0	12.2	27.7	60.1	39.9	-0.097	-0.388
	D	26.5	36.4	12.8	24.3	62.9	37.1	-0.157	-0.310
	E	28.3	33.1	10.9	27.7	61.4	38.6	-0.079	-0.433
	F	27.7	33.6	11.5	27.2	61.3	38.7	-0.096	-0.405
	G	29.0	34.4	10.6	26.0	63.4	36.6	-0.086	-0.421
	H	28.0	33.7	10.7	27.7	61.7	38.4	-0.093	-0.443
	I	25.5	35.5	11.4	27.6	61.0	39.0	-0.163	-0.414
COIII	A	27.6	32.0	16.7	23.7	59.6	40.4	-0.074	-0.173
	B	25.6	30.0	18.4	26.0	55.6	44.4	-0.079	-0.171
	C	27.2	31.1	16.8	24.9	58.3	41.7	-0.067	-0.194
	D	25.9	32.4	17.3	24.4	58.3	41.7	-0.111	-0.170
	E	27.4	32.1	16.5	24.0	59.5	40.5	-0.079	-0.186
	F	27.6	32.4	16.6	23.5	60.0	40.1	-0.081	-0.172
	G	27.8	33.4	16.1	22.7	61.2	38.8	-0.093	-0.169
	H	27.6	31.2	16.1	25.1	58.8	41.2	-0.061	-0.221
	I	26.0	30.0	17.2	26.8	56.0	44.0	-0.071	-0.217
ND3	A	25.0	32.4	16.8	25.9	57.4	42.7	-0.129	-0.213
	B	21.5	32.9	18.5	27.1	54.4	45.6	-0.210	-0.189
	C	22.6	31.8	17.9	27.6	54.4	45.5	-0.169	-0.212
	D	23.8	32.9	18.5	24.7	56.7	43.2	-0.160	-0.144
	E	25.7	35.0	15.5	23.9	60.7	39.4	-0.154	-0.215
	F	23.8	35.0	15.6	25.6	58.8	41.2	-0.191	-0.243
	G	24.7	36.8	16.5	22.1	61.5	38.6	-0.196	-0.145
	H	25.3	34.7	15.0	25.0	60.0	40.0	-0.157	-0.250
	I	23.9	32.1	14.0	30.0	56.0	44.0	-0.146	-0.364
ND4L	A	22.8	35.1	16.1	26.0	57.9	42.1	-0.212	-0.235
	B	23.9	30.9	16.8	28.4	54.8	45.2	-0.128	-0.257
	C	21.8	34.4	16.8	27.0	56.1	43.9	-0.225	-0.232
	D	26.0	31.6	14.7	27.7	57.6	42.4	-0.097	-0.307
	E	23.2	34.0	14.4	28.4	57.2	42.8	-0.191	-0.328
	F	27.4	36.1	11.9	24.6	63.5	36.5	-0.138	-0.346
	G	25.6	34.4	11.9	28.1	60.0	40.0	-0.146	-0.404
	H	26.7	36.5	11.2	25.6	63.2	36.8	-0.156	-0.392
	I	28.1	35.1	12.6	24.2	63.2	36.8	-0.111	-0.314

Table S4. *Cont.*

Region	Species	A%	T(U)%	G%	C%	A+T%	G+C%	AT skew	GC skew
	A	28.0	33.8	13.9	24.3	61.8	38.2	-0.094	-0.272
	B	28.0	33.9	14.0	24.1	61.9	38.1	-0.095	-0.265
	C	28.1	33.5	13.7	24.7	61.5	38.4	-0.087	-0.286
	D	28.0	34.2	14.5	23.4	62.2	37.9	-0.100	-0.235
ND4	E	29.5	32.8	12.5	25.2	62.3	37.7	-0.053	-0.339
	F	29.4	33.6	12.5	24.5	63.0	37.0	-0.066	-0.325
	G	29.2	34.0	12.2	24.6	63.2	36.8	-0.077	-0.337
	H	29.5	32.9	12.0	25.7	62.4	37.7	-0.054	-0.363
	I	26.3	31.8	14.1	27.7	58.1	41.8	-0.096	-0.325
	A	37.8	16.7	11.4	34.1	54.5	45.5	0.387	-0.499
	B	39.4	17.1	11.8	31.7	56.5	43.5	0.395	-0.457
	C	39.2	16.3	10.6	33.9	55.5	44.5	0.413	-0.524
	D	39.4	16.3	10.8	33.5	55.7	44.3	0.415	-0.512
ND6	E	40.7	13.9	11.9	33.5	54.6	45.4	0.491	-0.477
	F	39.8	20.7	12.0	27.4	60.5	39.4	0.315	-0.392
	G	43.8	17.0	9.8	29.4	60.8	39.2	0.441	-0.511
	H	43.6	18.2	9.4	28.8	61.8	38.2	0.411	-0.508
	I	37.8	19.3	10.4	32.5	57.1	42.9	0.324	-0.517
	A	26.7	30.4	14.5	28.3	57.1	42.8	-0.065	-0.322
	B	26.8	31.1	15.7	26.4	57.9	42.1	-0.074	-0.254
	C	27.8	31.5	14.0	26.7	59.2	40.7	-0.062	-0.312
	D	26.9	33.2	15.3	24.6	60.1	39.9	-0.105	-0.233
CYTB	E	27.3	27.5	14.2	31.0	54.8	45.2	-0.003	-0.371
	F	27.1	31.8	14.1	27.0	58.9	41.1	-0.079	-0.312
	G	29.0	29.3	13.3	28.4	58.3	41.7	-0.006	-0.363
	H	27.5	28.9	14.3	29.4	56.4	43.7	-0.025	-0.347
	I	25.6	27.8	15.1	31.5	53.4	46.6	-0.042	-0.352
	A	29.7	30.4	12.9	27.0	60.1	39.9	-0.012	-0.353
	B	30.4	28.8	12.6	28.2	59.2	40.8	0.027	-0.382
	C	30.2	28.6	12.7	28.5	58.8	41.2	0.029	-0.383
	D	28.7	30.8	14.1	26.4	59.5	40.5	-0.035	-0.304
ND5	E	31.3	27.0	11.8	29.8	58.3	41.6	0.073	-0.433
	F	31.8	30.8	13.3	24.1	62.6	37.4	0.015	-0.289
	G	32.3	30.0	11.6	26.1	62.3	37.7	0.037	-0.385
	H	31.4	28.4	12.1	28.2	59.8	40.3	0.052	-0.399
	I	29.8	32.4	12.7	25.1	62.2	37.8	-0.043	-0.326

A: *Polypedates impresus* (this study); B: *Polypedates mutus* (this study); C: *Polypedates megacephalus*; D: *Polypedates braueri*; E: *Zhangixalus schlegelii*; F: *Zhangixalus dennysi*; G: *Zhangixalus omeimontis*; H: *Zhangixalus arboreus*; I: *Buergeria buergeri*.
