# Supplementary material for: Complete Mitogenomes of Polypedates Tree Frogs Unveil Gene Rearrangement and Concerted Evolution within Rhacophoridae
Source: Animals (Basel). 2022 Sep 16;12(18):2449. doi: 10.3390/ani12182449 (PMC9494961; doi:10.3390/ani12182449)
Supplement: Supplementary file 1 [file animals-12-02449-s001.zip › Table S5. Start and Stop Codon.pdf]

Table S5. Mitogenome start and stop codon usage of nine rhacophorids.

PCGs	Start Codon									Stop Codon								
	A	B	C	D	E	F	G	H	I	A	B	C	D	E	F	G	H	I
ATP6	ATA	ATA	ATA	ATA	ATT	ATG	ATA	ATG	ATG	T(AA)	T(AA)	T(AA)	T(AA)	T(AA)	T(AA)	AGG	TA(A)	T(AA)
ATP8	N/A	N/A	N/A	N/A	ATG	ATG	ATG	ATG	ATG	N/A	N/A	N/A	N/A	TAA	TAA	TAA	TAA	TAA
COI	ATA	ATA	ATA	ATA	ATA	ATA	GTG	ATA	ATA	AGG	AGG	AGG	AGG	AGG	AGG	AGG	AGG	AGG
COII	ATA	ATG	ATA	ATG	ATG	ATG	ATG	ATG	ATG	TAA	TAA	TAA	TAA	TAA	TAA	TAG	TAA	AGA
COIII	ATG	ATG	ATG	ATG	ATG	ATG	ATA	ATG	ATG	T(AA)	T(AA)	T(AA)	T(AA)	T(AA)	T(AA)	T(AA)	TA(A)	T(AA)
CYTB	ATG	ATG	ATG	ATG	ATG	ATG	ATG	ATG	ATG	T(AA)	T(AA)	T(AA)	T(AA)	TAG	TAA	TAA	AGA	TAA
ND1	ATG	ATG	ATG	ATG	GTG	ATA	ATG	ATG	ATT	T(AA)	T(AA)	T(AA)	T(AA)	T(AA)	T(AA)	T(AA)	T(AA)	T(AA)
ND2	ATT	ATT	ATT	ATT	GTG	ATT	ATT	ATG	ATT	TAG	TAG	T(AA)	T(AA)	TAA	TAG	TAA	TAA	TAG
ND3	ATG	ATG	ATG	ATG	ATA	ATG	ATG	ATG	ATA	T(AA)	T(AA)	T(AA)	T(AA)	T(AA)	T(AA)	T(AA)	T(AA)	T(AA)
ND4	GTG	ATG	GTG	ATG	ATG	ATG	ATG	ATG	ATG	T(AA)	T(AA)	T(AA)	T(AA)	T(AA)	T(AA)	T(AA)	T(AA)	T(AA)
ND4L	ATG	ATG	ATG	ATG	ATG	ATG	ATG	ATG	ATG	TAA	TAA	TAA	TAA	TAG	TAA	TAA	TAG	TAA
ND5	ATG	ATG	ATG	ATG	ATG	ATG	ATA	ATG	ATG	TAA	TAA	TAA	TAG	TAG	TAA	TAA	AGA	AGA
ND6	ATG	ATG	ATG	ATG	ATG	ATG	ATG	ATG	ATG	AGG	AGG	AGG	AGG	AGA	AGG	AGA	AGA	AGG

A: *Polypedates impresus* (this study); B: *Polypedates mutus* (this study); C: *Polypedates megacephalus*; D: *Polypedates braueri*; E: *Zhangixalus schlegelii*; F: *Zhangixalus dennysi*; G: *Zhangixalus omeimontis*; H: *Zhangixalus arboreus*; I: *Buergeria buergeri*.
