# Supplementary material for: Complete Mitogenomes of Polypedates Tree Frogs Unveil Gene Rearrangement and Concerted Evolution within Rhacophoridae
Source: Animals (Basel). 2022 Sep 16;12(18):2449. doi: 10.3390/ani12182449 (PMC9494961; doi:10.3390/ani12182449)
Supplement: Supplementary file 1 [file animals-12-02449-s001.zip › Table S6. Codon Usage.pdf]

Table S6. Mitogenome codon usage of nine rhacophorids.

A *Polypedates impresus*				
**AA**	**Codon**	**Count**	**RSCU**	**AARatio**
Phe	UUU	171	1.29	
Phe	UUC	95	0.71	7.19%
Leu2	UUA	162	1.66	
Leu2	UUG	30	0.31	5.19%
Leu1	CUU	139	1.42	
Leu1	CUC	67	0.69	
Leu1	CUA	134	1.37	
Leu1	CUG	54	0.55	10.65%
Ile	AUU	237	1.52	
Ile	AUC	74	0.48	8.41%
Met	AUA	155	1.53	
Met	AUG	47	0.47	5.46%
Val	GUU	84	1.66	
Val	GUC	26	0.51	
Val	GUA	57	1.13	
Val	GUG	35	0.69	5.46%
Ser2	UCU	50	1.24	
Ser2	UCC	45	1.12	
Ser2	UCA	86	2.13	
Ser2	UCG	13	0.32	5.24%
Pro	CCU	38	0.81	
Pro	CCC	45	0.96	
Pro	CCA	91	1.94	
Pro	CCG	14	0.3	5.08%
Thr	ACU	73	0.98	
Thr	ACC	80	1.07	
Thr	ACA	123	1.65	
Thr	ACG	22	0.3	8.05%
Ala	GCU	71	0.87	
Ala	GCC	111	1.35	
Ala	GCA	124	1.51	
Ala	GCG	22	0.27	8.86%
Tyr	UAU	67	1.15	
Tyr	UAC	50	0.85	3.16%
His	CAU	45	0.88	
His	CAC	57	1.12	2.76%
Gln	CAA	70	1.71	
Gln	CAG	12	0.29	2.22%
Asn	AAU	63	0.95	
Asn	AAC	69	1.05	3.57%
Lys	AAA	76	1.73	
Lys	AAG	12	0.27	2.38%
Asp	GAU	25	0.74	
Asp	GAC	43	1.26	1.84%
Glu	GAA	47	1.18	
Glu	GAG	33	0.82	2.16%
Cys	UGU	12	0.92	
Cys	UGC	14	1.08	0.7%
Trp	UGA	84	1.66	
Trp	UGG	17	0.34	2.73%
Arg	CGU	10	0.61	
Arg	CGC	31	1.88	
Arg	CGA	19	1.15	
Arg	CGG	6	0.36	1.78%
Ser1	AGU	19	0.47	
Ser1	AGC	29	0.72	1.3%
Gly	GGU	34	0.63	
Gly	GGC	89	1.66	
Gly	GGA	61	1.13	
Gly	GGG	31	0.58	5.81%

Table S6. *Cont.*

B *Polypedates mutus*				
**AA**	**Codon**	**Count**	**RSCU**	**AARatio**
Phe	UUU	171	1.29	
Phe	UUC	95	0.71	7.19%
Leu2	UUA	178	1.81	
Leu2	UUG	34	0.35	5.73%
Leu1	CUU	122	1.24	
Leu1	CUC	83	0.85	
Leu1	CUA	127	1.29	
Leu1	CUG	45	0.46	10.19%
Ile	AUU	226	1.43	
Ile	AUC	89	0.57	8.52%
Met	AUA	145	1.42	
Met	AUG	59	0.58	5.52%
Val	GUU	58	1.23	
Val	GUC	32	0.68	
Val	GUA	70	1.48	
Val	GUG	29	0.61	5.11%
Ser2	UCU	41	1	
Ser2	UCC	57	1.39	
Ser2	UCA	91	2.22	
Ser2	UCG	10	0.24	5.38%
Pro	CCU	35	0.73	
Pro	CCC	41	0.86	
Pro	CCA	95	1.99	
Pro	CCG	20	0.42	5.16%
Thr	ACU	70	0.93	
Thr	ACC	85	1.13	
Thr	ACA	124	1.65	
Thr	ACG	21	0.28	8.11%
Ala	GCU	63	0.77	
Ala	GCC	122	1.5	
Ala	GCA	125	1.53	
Ala	GCG	16	0.2	8.82%
Tyr	UAU	64	1.15	
Tyr	UAC	47	0.85	3%
His	CAU	34	0.69	
His	CAC	65	1.31	2.68%
Gln	CAA	68	1.7	
Gln	CAG	12	0.3	2.16%
Asn	AAU	59	0.94	
Asn	AAC	67	1.06	3.41%
Lys	AAA	72	1.62	
Lys	AAG	17	0.38	2.41%
Asp	GAU	28	0.77	
Asp	GAC	45	1.23	1.97%
Glu	GAA	54	1.37	
Glu	GAG	25	0.63	2.14%
Cys	UGU	11	0.79	
Cys	UGC	17	1.21	0.76%
Trp	UGA	82	1.59	
Trp	UGG	21	0.41	2.79%
Arg	CGU	15	0.87	
Arg	CGC	17	0.99	
Arg	CGA	29	1.68	
Arg	CGG	8	0.46	1.87%
Ser1	AGU	18	0.44	
Ser1	AGC	29	0.71	1.27%
Gly	GGU	34	0.63	
Gly	GGC	84	1.56	
Gly	GGA	49	0.91	
Gly	GGG	48	0.89	5.81%

Table S6. *Cont.*

C *Polypedates megacephalus*				
**AA**	**Codon**	**Count**	**RSCU**	**AARatio**
Phe	UUU	167	1.27	
Phe	UUC	95	0.73	7.08%
Leu2	UUA	154	1.57	
Leu2	UUG	36	0.37	5.14%
Leu1	CUU	131	1.33	
Leu1	CUC	85	0.86	
Leu1	CUA	135	1.37	
Leu1	CUG	49	0.5	10.81%
Ile	AUU	221	1.43	
Ile	AUC	89	0.57	8.38%
Met	AUA	156	1.56	
Met	AUG	44	0.44	5.41%
Val	GUU	79	1.54	
Val	GUC	39	0.76	
Val	GUA	59	1.15	
Val	GUG	28	0.55	5.54%
Ser2	UCU	41	1.05	
Ser2	UCC	44	1.12	
Ser2	UCA	91	2.32	
Ser2	UCG	15	0.38	5.16%
Pro	CCU	41	0.85	
Pro	CCC	44	0.91	
Pro	CCA	92	1.91	
Pro	CCG	16	0.33	5.22%
Thr	ACU	70	0.93	
Thr	ACC	80	1.06	
Thr	ACA	128	1.7	
Thr	ACG	24	0.32	8.16%
Ala	GCU	76	0.93	
Ala	GCC	105	1.28	
Ala	GCA	131	1.6	
Ala	GCG	16	0.2	8.87%
Tyr	UAU	66	1.15	
Tyr	UAC	49	0.85	3.11%
His	CAU	36	0.72	
His	CAC	64	1.28	2.7%
Gln	CAA	66	1.63	
Gln	CAG	15	0.37	2.19%
Asn	AAU	59	0.94	
Asn	AAC	67	1.06	3.41%
Lys	AAA	72	1.66	
Lys	AAG	15	0.34	2.35%
Asp	GAU	27	0.76	
Asp	GAC	44	1.24	1.92%
Glu	GAA	54	1.37	
Glu	GAG	25	0.63	2.14%
Cys	UGU	10	0.77	
Cys	UGC	16	1.23	0.7%
Trp	UGA	84	1.66	
Trp	UGG	17	0.34	2.73%
Arg	CGU	10	0.57	
Arg	CGC	30	1.71	
Arg	CGA	22	1.26	
Arg	CGG	8	0.46	1.89%
Ser1	AGU	15	0.38	
Ser1	AGC	29	0.74	1.19%
Gly	GGU	32	0.59	
Gly	GGC	96	1.76	
Gly	GGA	53	0.97	
Gly	GGG	37	0.68	5.89%

Table S6. *Cont.*

D *Polypedates braueri*				
**AA**	**Codon**	**Count**	**RSCU**	**AARatio**
Phe	UUU	185	1.37	
Phe	UUC	86	0.63	7.33%
Leu2	UUA	183	1.88	
Leu2	UUG	38	0.39	5.98%
Leu1	CUU	120	1.23	
Leu1	CUC	69	0.71	
Leu1	CUA	116	1.19	
Leu1	CUG	59	0.61	9.84%
Ile	AUU	238	1.52	
Ile	AUC	75	0.48	8.46%
Met	AUA	141	1.46	
Met	AUG	52	0.54	5.22%
Val	GUU	73	1.37	
Val	GUC	32	0.6	
Val	GUA	73	1.37	
Val	GUG	35	0.66	5.76%
Ser2	UCU	52	1.21	
Ser2	UCC	48	1.12	
Ser2	UCA	86	2	
Ser2	UCG	18	0.42	5.52%
Pro	CCU	38	0.82	
Pro	CCC	28	0.6	
Pro	CCA	98	2.11	
Pro	CCG	22	0.47	5.03%
Thr	ACU	72	1.05	
Thr	ACC	80	1.17	
Thr	ACA	101	1.47	
Thr	ACG	21	0.31	7.41%
Ala	GCU	77	0.93	
Ala	GCC	105	1.27	
Ala	GCA	126	1.52	
Ala	GCG	24	0.29	8.98%
Tyr	UAU	72	1.29	
Tyr	UAC	40	0.71	3.03%
His	CAU	39	0.8	
His	CAC	58	1.2	2.62%
Gln	CAA	70	1.56	
Gln	CAG	20	0.44	2.43%
Asn	AAU	79	1.33	
Asn	AAC	40	0.67	3.22%
Lys	AAA	66	1.53	
Lys	AAG	20	0.47	2.33%
Asp	GAU	29	0.79	
Asp	GAC	44	1.21	1.97%
Glu	GAA	58	1.45	
Glu	GAG	22	0.55	2.16%
Cys	UGU	11	0.81	
Cys	UGC	16	1.19	0.73%
Trp	UGA	81	1.54	
Trp	UGG	24	0.46	2.84%
Arg	CGU	9	0.52	
Arg	CGC	24	1.39	
Arg	CGA	26	1.51	
Arg	CGG	10	0.58	1.87%
Ser1	AGU	19	0.44	
Ser1	AGC	35	0.81	1.46%
Gly	GGU	41	0.76	
Gly	GGC	90	1.67	
Gly	GGA	49	0.91	
Gly	GGG	35	0.65	5.81%

Table S6. *Cont.*

E *Zhangixalus schlegeli*				
**AA**	**Codon**	**Count**	**RSCU**	**AARatio**
Phe	UUU	167	1.3	
Phe	UUC	89	0.7	6.86%
Leu2	UUA	161	1.66	
Leu2	UUG	31	0.32	5.14%
Leu1	CUU	99	1.02	
Leu1	CUC	78	0.81	
Leu1	CUA	177	1.83	
Leu1	CUG	35	0.36	10.42%
Ile	AUU	211	1.33	
Ile	AUC	107	0.67	8.52%
Met	AUA	153	1.52	
Met	AUG	48	0.48	5.38%
Val	GUU	57	1.21	
Val	GUC	32	0.68	
Val	GUA	80	1.69	
Val	GUG	20	0.42	5.06%
Ser2	UCU	64	1.43	
Ser2	UCC	63	1.41	
Ser2	UCA	89	1.99	
Ser2	UCG	8	0.18	6%
Pro	CCU	30	0.61	
Pro	CCC	74	1.49	
Pro	CCA	87	1.76	
Pro	CCG	7	0.14	5.3%
Thr	ACU	65	0.87	
Thr	ACC	101	1.36	
Thr	ACA	121	1.62	
Thr	ACG	11	0.15	7.98%
Ala	GCU	73	0.93	
Ala	GCC	130	1.65	
Ala	GCA	100	1.27	
Ala	GCG	12	0.15	8.44%
Tyr	UAU	55	1.05	
Tyr	UAC	50	0.95	2.81%
His	CAU	36	0.71	
His	CAC	66	1.29	2.73%
Gln	CAA	79	1.8	
Gln	CAG	9	0.2	2.36%
Asn	AAU	49	0.72	
Asn	AAC	88	1.28	3.67%
Lys	AAA	79	1.72	
Lys	AAG	13	0.28	2.46%
Asp	GAU	29	0.82	
Asp	GAC	42	1.18	1.9%
Glu	GAA	63	1.5	
Glu	GAG	21	0.5	2.25%
Cys	UGU	12	0.77	
Cys	UGC	19	1.23	0.83%
Trp	UGA	91	1.64	
Trp	UGG	20	0.36	2.97%
Arg	CGU	8	0.45	
Arg	CGC	13	0.73	
Arg	CGA	45	2.54	
Arg	CGG	5	0.28	1.9%
Ser1	AGU	16	0.36	
Ser1	AGC	28	0.63	1.18%
Gly	GGU	33	0.61	
Gly	GGC	52	0.95	
Gly	GGA	89	1.63	
Gly	GGG	44	0.81	5.84%

Table S6. *Cont.*

F *Zhangixalus dennysi*				
**AA**	**Codon**	**Count**	**RSCU**	**AARatio**
Phe	UUU	168	1.34	
Phe	UUC	82	0.66	6.66%
Leu2	UUA	192	1.92	
Leu2	UUG	32	0.32	5.97
Leu1	CUU	115	1.15	
Leu1	CUC	76	0.76	
Leu1	CUA	162	1.62	
Leu1	CUG	22	0.22	9.99%
Ile	AUU	224	1.37	
Ile	AUC	104	0.63	8.74%
Met	AUA	151	1.5	
Met	AUG	50	0.5	5.36%
Val	GUU	52	1.2	
Val	GUC	31	0.71	
Val	GUA	74	1.7	
Val	GUG	17	0.39	4.64%
Ser2	UCU	59	1.25	
Ser2	UCC	67	1.42	
Ser2	UCA	100	2.11	
Ser2	UCG	8	0.17	6.24%
Pro	CCU	32	0.65	
Pro	CCC	58	1.18	
Pro	CCA	98	1.99	
Pro	CCG	9	0.18	5.25%
Thr	ACU	72	0.96	
Thr	ACC	99	1.32	
Thr	ACA	123	1.64	
Thr	ACG	6	0.08	8%
Ala	GCU	75	0.96	
Ala	GCC	123	1.57	
Ala	GCA	104	1.33	
Ala	GCG	11	0.14	8.34%
Tyr	UAU	63	1.16	
Tyr	UAC	46	0.84	2.91%
His	CAU	40	0.82	
His	CAC	58	1.18	2.61%
Gln	CAA	90	1.96	
Gln	CAG	2	0.04	2.45%
Asn	AAU	58	0.82	
Asn	AAC	83	1.18	3.76%
Lys	AAA	75	1.7	
Lys	AAG	13	0.3	2.35%
Asp	GAU	31	0.9	
Asp	GAC	38	1.1	1.84%
Glu	GAA	71	1.65	
Glu	GAG	15	0.35	2.29%
Cys	UGU	19	1.27	
Cys	UGC	11	0.73	0.8%
Trp	UGA	96	1.73	
Trp	UGG	15	0.27	2.96%
Arg	CGU	8	0.46	
Arg	CGC	11	0.64	
Arg	CGA	46	2.67	
Arg	CGG	4	0.23	1.84%
Ser1	AGU	22	0.46	
Ser1	AGC	28	0.59	1.33%
Gly	GGU	43	0.81	
Gly	GGC	50	0.94	
Gly	GGA	79	1.48	
Gly	GGG	41	0.77	5.68%

Table S6. *Cont.*

G *Zhangixalus omeimontis*				
**AA**	**Codon**	**Count**	**RSCU**	**AARatio**
Phe	UUU	173	1.36	
Phe	UUC	82	0.64	6.72%
Leu2	UUA	196	2.01	
Leu2	UUG	22	0.23	5.75%
Leu1	CUU	129	1.32	
Leu1	CUC	51	0.52	
Leu1	CUA	159	1.63	
Leu1	CUG	28	0.29	9.68%
Ile	AUU	247	1.44	
Ile	AUC	97	0.56	9.07%
Met	AUA	185	1.57	
Met	AUG	51	0.43	6.22%
Val	GUU	58	1.27	
Val	GUC	27	0.59	
Val	GUA	75	1.65	
Val	GUG	22	0.48	4.8%
Ser2	UCU	64	1.37	
Ser2	UCC	62	1.32	
Ser2	UCA	104	2.22	
Ser2	UCG	6	0.13	6.22%
Pro	CCU	33	0.67	
Pro	CCC	61	1.24	
Pro	CCA	98	1.99	
Pro	CCG	5	0.1	5.2%
Thr	ACU	88	1.21	
Thr	ACC	78	1.07	
Thr	ACA	116	1.59	
Thr	ACG	9	0.12	7.67%
Ala	GCU	78	1.03	
Ala	GCC	117	1.55	
Ala	GCA	103	1.36	
Ala	GCG	4	0.05	7.96%
Tyr	UAU	67	1.12	
Tyr	UAC	53	0.88	3.16%
His	CAU	50	1.01	
His	CAC	49	0.99	2.61%
Gln	CAA	80	1.88	
Gln	CAG	5	0.12	2.24%
Asn	AAU	75	1.04	
Asn	AAC	69	0.96	3.8%
Lys	AAA	79	1.72	
Lys	AAG	13	0.28	2.43%
Asp	GAU	37	1.01	
Asp	GAC	36	0.99	1.93%
Glu	GAA	64	1.58	
Glu	GAG	17	0.42	2.14%
Cys	UGU	20	1.33	
Cys	UGC	10	0.67	0.79%
Trp	UGA	94	1.71	
Trp	UGG	16	0.29	2.9%
Arg	CGU	11	0.62	
Arg	CGC	10	0.56	
Arg	CGA	45	2.54	
Arg	CGG	5	0.28	1.87%
Ser1	AGU	18	0.38	
Ser1	AGC	27	0.58	1.19%
Gly	GGU	37	0.69	
Gly	GGC	50	0.93	
Gly	GGA	85	1.59	
Gly	GGG	42	0.79	5.64%

Table S6. *Cont.*

H *Zhangixalus arboreus*				
**AA**	**Codon**	**Count**	**RSCU**	**AARatio**
Phe	UUU	168	1.34	
Phe	UUC	82	0.66	6.66%
Leu2	UUA	192	1.92	
Leu2	UUG	32	0.32	5.97
Leu1	CUU	115	1.15	
Leu1	CUC	76	0.76	
Leu1	CUA	162	1.62	
Leu1	CUG	22	0.22	9.99%
Ile	AUU	224	1.37	
Ile	AUC	104	0.63	8.74%
Met	AUA	151	1.5	
Met	AUG	50	0.5	5.36%
Val	GUU	52	1.2	
Val	GUC	31	0.71	
Val	GUA	74	1.7	
Val	GUG	17	0.39	4.64%
Ser2	UCU	59	1.25	
Ser2	UCC	67	1.42	
Ser2	UCA	100	2.11	
Ser2	UCG	8	0.17	6.24%
Pro	CCU	32	0.65	
Pro	CCC	58	1.18	
Pro	CCA	98	1.99	
Pro	CCG	9	0.18	5.25%
Thr	ACU	72	0.96	
Thr	ACC	99	1.32	
Thr	ACA	123	1.64	
Thr	ACG	6	0.08	8%
Ala	GCU	75	0.96	
Ala	GCC	123	1.57	
Ala	GCA	104	1.33	
Ala	GCG	11	0.14	8.34%
Tyr	UAU	63	1.16	
Tyr	UAC	46	0.84	2.91%
His	CAU	40	0.82	
His	CAC	58	1.18	2.61%
Gln	CAA	90	1.96	
Gln	CAG	2	0.04	2.45%
Asn	AAU	58	0.82	
Asn	AAC	83	1.18	3.76%
Lys	AAA	75	1.7	
Lys	AAG	13	0.3	2.35%
Asp	GAU	31	0.9	
Asp	GAC	38	1.1	1.84%
Glu	GAA	71	1.65	
Glu	GAG	15	0.35	2.29%
Cys	UGU	19	1.27	
Cys	UGC	11	0.73	0.8%
Trp	UGA	96	1.73	
Trp	UGG	15	0.27	2.96%
Arg	CGU	8	0.46	
Arg	CGC	11	0.64	
Arg	CGA	46	2.67	
Arg	CGG	4	0.23	1.84%
Ser1	AGU	22	0.46	
Ser1	AGC	28	0.59	1.33%
Gly	GGU	43	0.81	
Gly	GGC	50	0.94	
Gly	GGA	79	1.48	
Gly	GGG	41	0.77	5.68%

Table S6. *Cont.*

I *Buergeria buergeri*				
**AA**	**Codon**	**Count**	**RSCU**	**AARatio**
Phe	UUU	158	1.26	
Phe	UUC	92	0.74	6.64%
Leu2	UUA	138	1.43	
Leu2	UUG	28	0.29	4.41%
Leu1	CUU	130	1.35	
Leu1	CUC	102	1.06	
Leu1	CUA	140	1.45	
Leu1	CUG	41	0.42	10.98%
Ile	AUU	206	1.28	
Ile	AUC	115	0.72	8.53%
Met	AUA	152	1.59	
Met	AUG	39	0.41	5.08%
Val	GUU	69	1.33	
Val	GUC	52	1	
Val	GUA	58	1.12	
Val	GUG	29	0.56	5.53%
Ser2	UCU	79	1.74	
Ser2	UCC	59	1.3	
Ser2	UCA	82	1.8	
Ser2	UCG	11	0.24	6.14%
Pro	CCU	41	0.84	
Pro	CCC	56	1.14	
Pro	CCA	72	1.47	
Pro	CCG	27	0.55	5.21%
Thr	ACU	95	1.26	
Thr	ACC	79	1.05	
Thr	ACA	112	1.48	
Thr	ACG	16	0.21	8.03%
Ala	GCU	99	1.21	
Ala	GCC	126	1.54	
Ala	GCA	95	1.16	
Ala	GCG	8	0.1	8.72%
Tyr	UAU	60	1.03	
Tyr	UAC	56	0.97	3.08%
His	CAU	32	0.65	
His	CAC	66	1.35	2.6%
Gln	CAA	88	1.83	
Gln	CAG	8	0.17	2.55%
Asn	AAU	64	0.96	
Asn	AAC	69	1.04	3.53%
Lys	AAA	79	1.76	
Lys	AAG	11	0.24	2.39%
Asp	GAU	27	0.77	
Asp	GAC	43	1.23	1.86%
Glu	GAA	71	1.58	
Glu	GAG	19	0.42	2.39%
Cys	UGU	15	1.07	
Cys	UGC	13	0.93	0.74%
Trp	UGA	93	1.71	
Trp	UGG	16	0.29	2.9%
Arg	CGU	15	0.81	
Arg	CGC	18	0.97	
Arg	CGA	38	2.05	
Arg	CGG	3	0.16	1.97%
Ser1	AGU	15	0.33	
Ser1	AGC	27	0.59	1.12%
Gly	GGU	39	0.74	
Gly	GGC	58	1.1	
Gly	GGA	71	1.35	
Gly	GGG	43	0.82	5.61%
