# Supplementary figures and images for: Complete Mitogenomes of Polypedates Tree Frogs Unveil Gene Rearrangement and Concerted Evolution within Rhacophoridae
Source: Animals (Basel). 2022 Sep 16;12(18):2449. doi: 10.3390/ani12182449 (PMC9494961; doi:10.3390/ani12182449)

Tree scale: 0.1

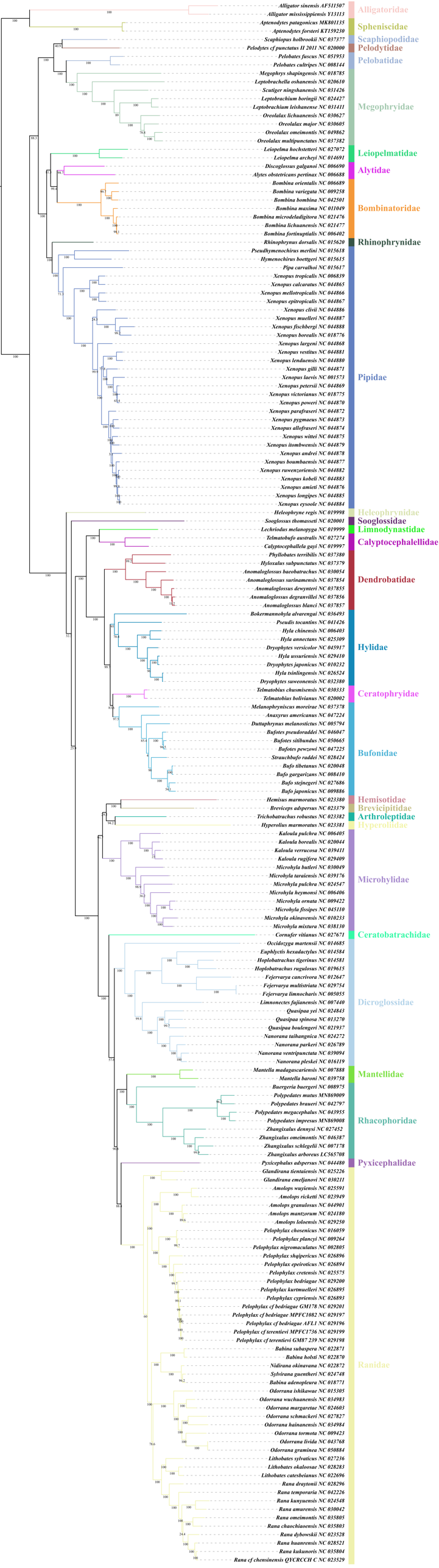

Supplement: Supplementary file 1 [file animals-12-02449-s001.zip › Figure S1. Phylogram ML.pdf]

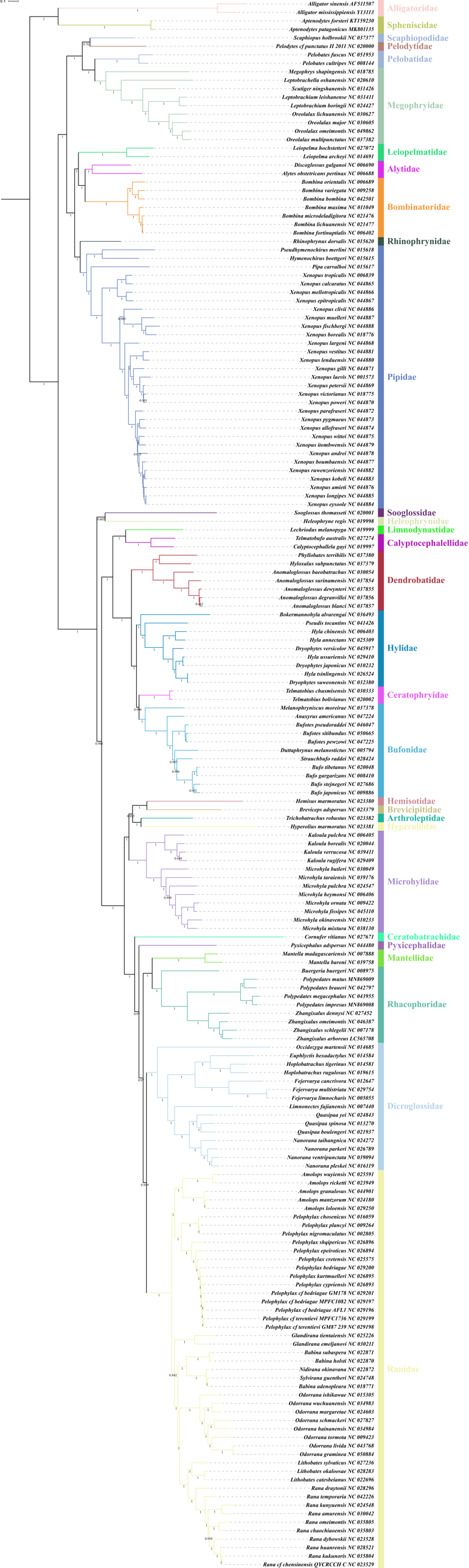

Supplement: Supplementary file 1 [file animals-12-02449-s001.zip › Figure S2. Phylogram BI.pdf]
